# Systematic review and meta‐analysis of randomized controlled trials of psychological interventions to improve glycaemic control in children and adults with type 1 diabetes

**DOI:** 10.1111/dme.14264

**Published:** 2020-03-10

**Authors:** K. Winkley, R. Upsher, D. Stahl, D. Pollard, A. Brennan, S. Heller, K. Ismail

**Affiliations:** ^1^ Florence Nightingale Faculty of Nursing, Midwifery & Palliative Care London UK; ^2^ Department of Psychological Medicine Institute of Psychiatry, Psychology and Neuroscience London UK; ^3^ Department of Biostatistics Institute of Psychiatry King's College London London UK; ^4^ School of Health and Related Research University of Sheffield Sheffield UK; ^5^ Department of Oncology & Metabolism University of Sheffield School of Medicine Sheffield UK

## Abstract

**Aim:**

We conducted a systematic review aggregate and network meta‐analysis of psychological interventions for people with type 1 diabetes to assess their effectiveness in improving glycaemic levels.

**Methods:**

We searched the following databases from 1 January 2003 to 1 July 2018: MEDLINE, CINAHL, PsycINFO, Embase, Cochrane Controlled Trials, Web of Science, https://clinicaltrials.gov, Dissertation Abstract International. We included randomized controlled trials (RCT) of psychological interventions for children and adults with type 1 diabetes reported in any language. We extracted data on publications, participant characteristics at baseline, intervention and control group, and data for the primary outcome, change in glycaemic control [HbA_1c_ (mmol/mol/%)]. Study authors were contacted for missing data. The review was registered with international prospective register of systematic reviews registration (PROSPERO) CRD42016033619.

**Results:**

Twenty‐four adult RCTs and 23 of children with type 1 diabetes were included in the systematic review. In aggregate meta‐analysis there was no overall effect of psychological intervention compared with control on HbA_1c_ [adults, nine RCTs, *n* = 1102, pooled mean difference −0.12, 95% confidence intervals (CI) −0.27 to 0.03, *I* ^2^ = 29.0%, *P* = 0.19; children, 20 RCTs, *n* = 2567, −0.09, 95% CI −0.22 to 0.04, *I* ^2^=54.0% *P*=0.002]. Network meta‐analysis suggested that probability and rank‐ordering of effectiveness is highest for attention control groups (*b* = −0.47, 95% CI −0.80 to −0.12) followed by cognitive behavioural therapy (CBT) (−0.26, 95% CI −0.45 to −0.06) compared with usual care for adults.

**Conclusions:**

Overall psychological interventions for children and adults with type 1 diabetes do not improve glycaemic control. For adults, CBT‐based interventions have the potential to be effective.

## What's new?

- A previous systematic review and meta‐analysis of randomized controlled trials (RCT) of psychological interventions to improve glycaemic control demonstrated an effect for children with type 1 diabetes but not adults, with a reduction in HbA_1c_ of 5 mmol/mol (0.5%).
- The current review synthesized data from nine adult (*n* = 1102) and 20 child RCTs (*n* = 2567) in an aggregate meta‐analysis; there was no improvement/reduction in HbA_1c_ for children or adults.
- Network meta‐analysis for adults demonstrated that cognitive behavioural therapy (CBT) and attention control were associated with statistically and clinically significant HbA_1c_ reduction.
- Psychological interventions are not indicated for improving glycaemic control for people with type 1 diabetes. For adults, CBT‐based interventions have the potential to be effective.

**What's new?**

- A previous systematic review and meta‐analysis of randomized controlled trials (RCT) of psychological interventions to improve glycaemic control demonstrated an effect for children with type 1 diabetes but not adults, with a reduction in HbA_1c_ of 5 mmol/mol (0.5%).
- The current review synthesized data from nine adult (*n* = 1102) and 20 child RCTs (*n* = 2567) in an aggregate meta‐analysis; there was no improvement/reduction in HbA_1c_ for children or adults.
- Network meta‐analysis for adults demonstrated that cognitive behavioural therapy (CBT) and attention control were associated with statistically and clinically significant HbA_1c_ reduction.
- Psychological interventions are not indicated for improving glycaemic control for people with type 1 diabetes. For adults, CBT‐based interventions have the potential to be effective.

## Introduction

Successful self‐management of type 1 diabetes involves the acquisition, implementation and maintenance of complex skills including frequent blood glucose monitoring, carbohydrate counting and calculations of insulin dose to achieve optimal glycaemic control and avoid diabetes‐related complications. Although emerging technologies are increasingly available to assist people with type 1 diabetes, self‐management remains highly complex and psychologically demanding. It is associated with high levels of psychological comorbidity, such as anxiety 1, depression 2 and eating disorders 3, and these problems are more prevalent in adults and children with diabetes and suboptimal glycaemic control 4, 5.

International guidelines 6, 7, 8, suggest that children and adults with type 1 diabetes should receive screening and psychological support in order to treat common psychological problems and relieve the daily stress of diabetes self‐management. Access to a range of mental health specialists is recommended to support children, their families and the diabetes team. Evidence suggests that for children, families and adults with type 1 diabetes, psychological support is important at the time of diagnosis to enable adjustment to the condition 9, 10. However, what type of psychological intervention should be delivered in terms of the psychological techniques or underlying psychological theory is open to debate. Interventions for adults and children are likely to be different, as for children these will most often involve the family group, and there may be specific underlying problems that need to be addressed, such as hyperglycaemia, hypoglycaemia unawareness 11, fear of hypoglycaemia 12 and fear of complications 13.

Our previous systematic review and meta‐analysis 14 of psychological interventions for children/adolescents and adults with type 1 diabetes demonstrated that psychological interventions were effective for improving glycaemic control for children, but not adults. As this was published more than 15 years ago we wanted to update it because: (1) different psychological treatments are being used and tested; (2) the statistical methods for synthesizing data across trials have progressed (such as using network meta‐analytic techniques which can simultaneously analyse multiple treatments across studies); and (3) most studies include glycaemic control to assess diabetes self‐management, whereas psychological outcomes vary across studies. Therefore, we considered the time was right to update our previous review and conduct a systematic review, aggregate and network‐meta‐analysis of RCTs to determine the effectiveness of psychological interventions in improving glycaemic control for children/adolescents and adults with type 1 diabetes.

## Methods

This systematic review and meta‐analysis followed the same protocol as for our earlier published review 14. The main outcome was change in HbA_1c_ (mmol/mol/%). We made a number of minor changes to the protocol such as additional details of the intervention in accordance with frameworks to improve intervention description and potential for replication 15. Network meta‐analysis was added to maximize the information gained from multiple treatment arms and control groups and comparisons, our updated protocol was published 16, and registered with the international prospective register of systematic reviews registration (PROSPERO) CRD42016033619. We employed the Cochrane risk of bias tool to assess study quality 17. We followed the Preferred Reporting Items for Systematic Reviews and Meta‐Analyses (PRISMA) statement 18.

### Eligibility criteria

Inclusion criteria were RCTs of a psychological intervention for children or adolescents or adults with type 1 diabetes. In brief, psychological interventions were defined as utilizing the therapeutic alliance between the individual(s) and therapist, facilitated by psychologists, psychotherapists or therapists, facilitated or supervised by the same, where the intervention was based on a psychological model and aimed to improve self‐management. Studies were reported in any language. We categorized psychological interventions as follows: (1) supportive or counselling therapy, including motivational interviewing; (2) cognitive behavioural therapy (CBT), including techniques commonly used in CBT such as relaxation, cognitive re‐structuring, goal‐setting, problem‐solving; (3) psychodynamic or interpersonal psychotherapy; and (4) family systems therapy. Newer therapies that may not fall into these categories, or interventions that did not explicitly describe the intervention or techniques underwent consensus discussion by an academic liaison psychiatrist, health psychologist and therapist trained in motivational interviewing (KI, RU, KW respectively). If agreement still could not be reached, they were excluded. We defined comparators as usual care, waiting list, and attention control (matched the number of sessions in the intervention arm) and diabetes education.

The main outcome was change in glycaemic control using HbA_1c_ (mmol/mol or %) between baseline and follow‐up, closest to 12 months.

### Information sources

We searched the following online databases from 1 January 2003 to 1 July 2018: MEDLINE (OVID), CINAHL, PsycINFO, Embase (OVID), Cochrane Controlled Trials Database, Web of Science, https://clinicaltrials.gov and Dissertation Abstracts International. We searched conference proceedings for a 5‐year period (2012 to 2018): Diabetes UK, American Diabetes Association, European Association for the Study of Diabetes and International Diabetes Federation. In addition to our earlier systematic review protocol we checked Web of Science, https://clinicaltrials.gov and Dissertation Abstracts International as these have become leading repositories over the last 15 years. We also checked reference lists of included studies and other reviews, leading authors, experts and investigators of ongoing RCTs were contacted.

### Search

The Cochrane collaboration's optimum search strategy was used and ‘diabetes mellitus’, ‘psychological therapies’ and ‘mood disorders’, and ‘clinical trials’ were used to search MEDLINE and adjusted for other databases (see Table S1).

### Study selection

Title and abstract screening of articles arising from the search was conducted by two independent reviewers (RU and KW) to determine whether articles met the inclusion criteria. Inter‐rater reliability was conducted to determine agreement for inclusion of studies. Studies were included if there was a disagreement. We excluded quasi RCT, N‐of‐1 and any study design other than RCTs.

### Data collection and data items

Full‐text review was conducted by both reviewers who independently extracted study data; non‐English studies were translated and data was extracted by a native speaker. Any disagreements regarding final inclusion were discussed with a third reviewer (KI) until consensus was reached. For studies with multiple publications, we included the one with data at baseline and follow‐up nearest to 12 months. For aggregate meta‐analysis, we included data from the most intensive psychological intervention if there were multiple treatments and included data from all intervention and control treatment arms in the network meta‐analysis. We requested missing data from study authors. Data was extracted on publication status such as country of origin and year. Participants characteristics included: age, gender, ethnicity, glycaemic control at baseline, duration of type 1 diabetes, and type of diabetes treatment. When studies included people with both type 1 and type 2 diabetes, only data on type 1 diabetes was extracted if findings had been stratified. Intervention characteristics were coded as type, duration, number of sessions, mode of delivery (individual, group, family), therapist characteristics (profession), manualized treatment, duration of follow up and follow‐up HbA_1c_. The intensity of the psychological intervention was determined by the number and duration of sessions (h) and the duration of the intervention (months). Data were extracted on the psychological theory underpinning the intervention, fidelity to the intervention and training and competency of the therapist.

### Statistical analysis and synthesis of results

To determine study quality, the Cochrane risk of bias (RoB) tool 17 was used independently by RU and KW to determine, high, low or unclear RoB both within and between studies. Disagreement was resolved by a third researcher. Subgroup meta‐analysis was conducted according to RoB rating, and we used meta‐regression to compare the effect sizes between RoB groups.

In aggregate meta‐analysis (combining data from one treatment and control arm across studies) the standardized mean difference (SMD), Cohen's *d*, was calculated to determine change in HbA_1c_ (mmol/mol or %) between baseline and 12‐month follow‐up or closest to that data point. Random effect meta‐analysis was used to pool SMDs. We calculated absolute HbA_1c_ values from the pooled sd and multiplied it by the overall SMD. The following diagnostic analyses were conducted: the effect of removing individual studies; Egger's publication bias; and funnel plots 19, trim and fill 20, to determine potential for missing studies. We conducted meta‐regression if there were five or more studies with data that could be pooled 21. Meta‐analyses were conducted using STATA 14 (StataCorp, College Station, TX, USA).

We combined the data from our previous meta‐analysis with that of the current one to determine cohort effects after removing any duplicate studies.

The network meta‐analysis involved analysis of direct and indirect effects of all of the treatment and control arms included in each study on the mean change in HbA_1c_ 22. Indirect effects compared categories of intervention (psychological interventions, alternative treatments) or control groups (usual care, attention control, waiting list, diabetes education) within and across studies. Network plots were constructed to show direct comparisons, we included contrasts in which there were at least two study sites contributing data. Random effects meta‐analysis allowing for heterogeneity and inconsistency between the studies was conducted 23. We compared direct and indirect effects of the contrast I‐J and Wald tests to determine inconsistency 24. Hedges’ *g* formula was used to determine unbiased SMDs corrected for degrees of freedom for different categories of intervention with usual care as the control. Potential ranks for each category were estimated using cumulative probability plots and surface under the cumulative ranking (SUCRA), the higher SUCRA (closest to one) the greater probability the intervention is effective.

## Results

### Study selection

**Figure 1:**
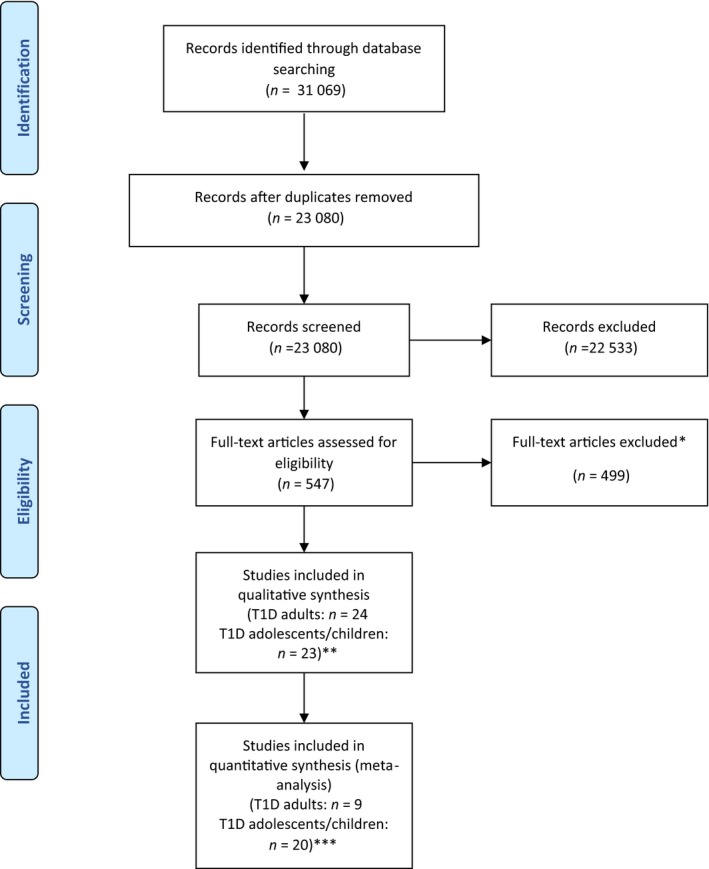
Qualitative and quantitative flowchart for all type 1 diabetes studies. *Reasons for exclusion: protocol (*n* = 41), conference abstracts (*n* = 37), outcome of interest reported in separate paper (*n* = 22), intervention not defined as psychological (*n* = 215), glycaemic control not measured (*n* = 47), not RCT (*n* = 25), unable to access study (*n* = 10), no diabetes (*n* = 8), type 2 diabetes (*n* = 95). **Fourteen type 1 diabetes adult studies and two type 1 diabetes child/adolescent studies were papers that included populations with type 1 and type 2 diabetes where separate analysis per diabetes type could not be obtained. The remaining single type 1 diabetes adult study and two type 1 diabetes child/adolescent studies that were not included in meta‐analysis, did not provide enough information for meta‐analysis. ***Three type 1 diabetes adult studies had populations with type 1 and type 2 diabetes where separate analysis per diabetes type was obtained.

We identified 31 609 study citations (Fig. 1). Once duplicates were removed, 23 080 citations underwent title and abstract screening; 547 full text articles were selected for further extraction. There was 94.5% agreement for identifying abstracts for full retrieval (Cohen's kappa 0.95). Twenty‐four adult and 23 child/adolescent type 1 diabetes RCTs met inclusion criteria for the systematic review. Reasons for the exclusion of the other studies are given in Fig. 1.

### Study characteristics

The studies included in the systematic review are listed and synthesized in Tables S2 and S3.

There was a broad range of clinical settings and/or criteria and for adult studies included in the systematic review, these included diabetes duration (*n* = 9) [S1–S9], age (*n* = 21) [S1–S5,S7–S22], suboptimal HbA_1c_ (*n* = 10) [S1,S2,S4–S6,S8,S9,S16,S22,S23], depressive symptoms (*n* = 4) [S7,S12,S16,S24] and emotional well‐being (*n* = 1) [S20]. There were no adult studies delivering psychodynamic, interpersonal therapy or family therapy, 12 studies delivered counselling [S4,S5,S9–S11,S15,S17,S18,S21–S24] and 12 CBT [S1–S3,S6–S8,S12–S14,S16,S19,S20]. In the control group, there were 11, 6 and 7 studies, administering usual care [S2,S4,S10,S14–S18,S20,S21,S24], attention control [S5–S8,S12,S23] or waiting list control [S1,S3,S9,S11,S13,S19,S22], respectively. Most therapists were diabetes specialists (*n* = 12) [S1,S2,S4,S8–S10,S13,S14,S17,S21–S23], psychology professionals (*n* = 9) [S3,S5–S7,S11,S12,S16,S19,S20] and ‘other’ (*n* = 3), defined as non‐diabetes health professionals (*n* = 2) [S15,S24] and peers (*n* = 1) [S18]. Most interventions were delivered face‐to‐face (*n* = 21) [S1–S9,S11–S17,S19,S20,S22–S24], via telephone (*n* = 2) [S10,S18], face‐to‐face and telephone (*n* = 1) [S21], and mostly to groups (*n* = 12) [S1,S4,S6,S8,S9,S11–S13,S16,S17,S20,S22] or one‐to‐one (*n* = 12) [S2,S3,S5,S7,S10,S14,S15,S18,S19,S21,S23,S24]. The mean number of therapy sessions offered was 7.68 (sd 2.67); the mean duration of each session was 1.58 h (sd 0.60); and mean duration of therapy was 5.3 months (sd 5.02). For adult studies, nine referred to an intervention manual [S1,S2,S5–S8,S11,S14,S16] of which four provided a link to the manual [S2,S7,S14,S16], and nine studies provided a link to the study protocol [S2,S3,S6,S7,S11,S14,S16,S17,S21].

For child/adolescent studies included in the systematic review, clinical settings and/or criteria included diabetes duration (*n* = 20) [S25–S44], age (*n* = 22) [S25–S34,S36–S43,S45–S47] and suboptimal HbA_1c_ (*n* = 9) [S26–S28,S30,S35,S38,S40,S42,S44]. There were no child/adolescent studies delivering a psychodynamic or interpersonal therapy, nine delivered counselling [S25,S26,S32,S33,S39–S42,S45], eight CBT [S29,S31,S34,S36,S37,S43,S46,S47] and six family systems therapy [S27,S28,S30,S35,S38,S44]. In the control group, there were 10, 11 and 2 studies administering usual care [S26,S28,S32,S34,S37,S38,S41,S44,S45,S47], attention control [S25,S27,S29–S31,S33,S36,S39,S40,S42,S43] or waiting list control [S35,S46], respectively. Therapists were diabetes specialists (*n* = 5) [S25,S26,S32,S42,S45], psychology professionals (*n* = 10) [S27–S30,S35,S37,S43,S44,S46,S47] and ‘other’ [defined as research assistants, non‐diabetes health professionals (*n* = 8)] [S31,S33,S34,S36,S38–S41]. Most interventions were delivered face‐to‐face (*n* = 18) [S25‐S32,S34,S37,S40–S47], via telephone (*n* = 3) [S33,S35,S36], face‐to‐face and telephone (*n* = 2) [S38,S39], and to groups (*n* = 6) [S26,S29,S42,S45–S47] or one‐to‐one (*n* = 5) [S25,S32,S36,S41,S43] or family (*n* = 12) [S27,S28,S30,S31,S33‐S35,S37–S40,S44]. The mean number of therapy sessions offered was 7.84 (sd 7.30); the mean duration of each session was 1.24 h (sd 0.84); and the mean duration of therapy was 9.02 months (sd 7.30). For child/adolescent studies, five referred to an intervention manual [S26,S39,S42,S44,S47] of which three provided a link to the manual [S26,S39,S47]; eight studies provided a link to the study protocol [S26–S29,S32,S33,S35,S36].

### Synthesis of results

**Figure 2:**
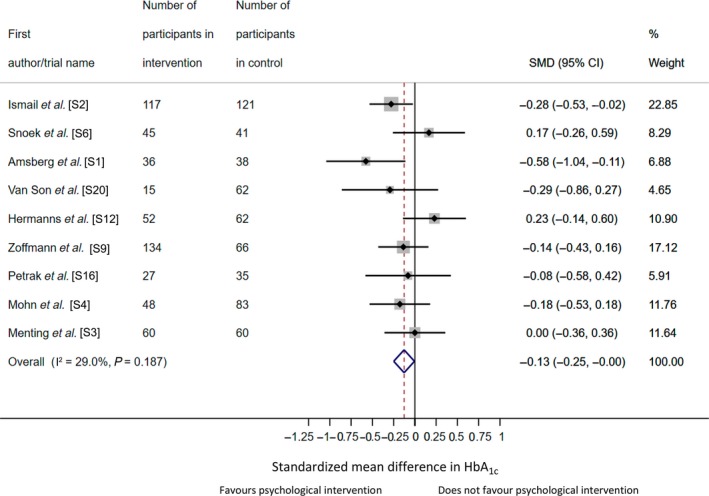
Forest plot for a random‐effects meta‐analysis of standardized mean difference in HbA_1c_ comparing psychological intervention vs. control group for adults with type 1 diabetes. References can be found in the online Supporting Information (Doc. S1).

There were nine adult studies with HbA_1c_ data to be pooled, giving a total sample of *n* = 1102. In the random effects meta‐analysis there was a non‐statistically significant reduction in HbA_1c_ for psychological intervention compared with the control group [SMD −0.12, 95% confidence intervals (CI) −0.27 to 0.03), equivalent to 2 mmol/mol (0.2%) reduction in HbA_1c_ (Fig. 2). Heterogeneity was low and non‐significant (*I* ^2^ = 29.0%, *P* = 0.19). When Snoek *et al*. [S6] or Hermanns *et al*. [S12] was removed as part of diagnostic analyses to determine the influence of individual studies, there was a significant decrease in HbA_1c_ in favour of psychological intervention (SMD −0.15, 95% CI −0.30 to −0.0002; SMD −0.17, 95% CI −0.30 to −0.04, respectively), both equivalent to reduction in HbA_1c_ of 2 mmol/mol (0.2%). There was no evidence of publication bias (Egger's test, *P* = 0.87). No additional studies were considered missing using trim and fill method. For adult studies, the majority of studies included a 12‐month follow‐up (*n* = 6) and follow‐up ranged between 5 and 48 months.

To examine whether there was a cohort effect, we pooled the HbA_1c_ data from 11 adult RCTs included in an earlier meta‐analysis (from inception to January 2004) with the current review (January 2003 to July 2018), totalling 20 RCTs (*n* = 1618). We derived a similar effect size to the current review (SMD −0.12, 95% CI −0.26 to 0.02), with no significant difference in effect sizes between reviews (*b* = −0.004, 95% CI −0.36 to 0.35, *P* = 0.98).

**Figure 3:**
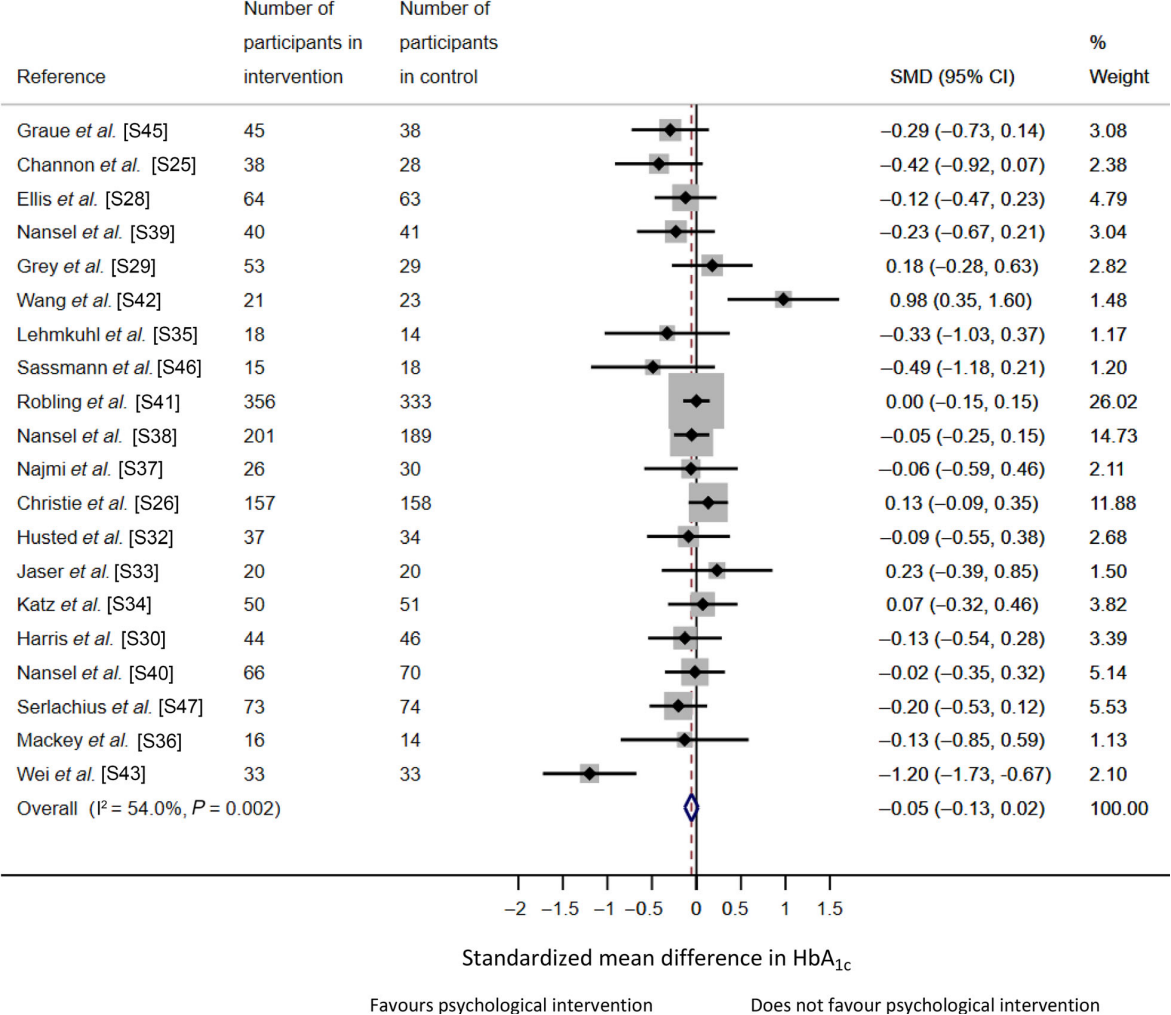
Forest plot for a random‐effects meta‐analysis of standardized mean difference in HbA_1c_ comparing psychological intervention vs. control group for children and adolescents with type 1 diabetes. References can be found in the online Supporting Information (Doc. S1).

For children/adolescents, 20 studies had HbA_1c_ data to be pooled giving a total sample of *n* = 2567. In the random effects meta‐analysis, there was a non‐statistically significant reduction in HbA_1c_ for psychological intervention compared with the control group (SMD −0.09, 95% CI −0.22 to 0.04), equivalent to a 1mmol/mol (0.1%) reduction in HbA_1c_ (Fig. 3). Heterogeneity was moderate and significant (*I* ^2^ = 54.0%, *P* = 0.002). There was no evidence of publication bias (Egger's test, *P* = 0.30), and no missing studies were detected. For child/adolescent studies, the majority included a 12‐month follow‐up (n = 12) and follow‐up ranged from 3 to 18 months.

To examine whether there was a cohort effect, we pooled the HbA_1c_ data from 10 child/adolescent RCTs included in an earlier meta‐analysis (from inception to January 2004) with the current review (January 2003 to July 2018), giving a total of 28 RCTs (*n* = 3018). We derived a similar effect size to the current review (SMD −0.11, 95% CI −0.29 to 0.06), with no significant difference in effect sizes between reviews (*b* = −0.27, 95% CI −1.05 to 0.51, *P* = 0.48).

### Risk of bias across studies

For adults, four studies were rated ‘unclear’ RoB and five studies were rated ‘low’ RoB (Fig. S1). The domains which were most ‘unclear’ risk were ‘random sequence generation’ and ‘blinding of participants and personnel’ (Fig. S2).

For children/adolescents, four studies were rated ‘low’ RoB and 16 ‘unclear’ RoB (Fig. S3). Between studies, ‘blinding of participants and personnel’ was rated most ‘unclear’ (Fig. S4).

### Additional analyses

For the meta‐regression for adults, there was no association between number of sessions (*b* = −0.03, 95% CI −0.09 to 0.02, *P* = 0.20), or session duration (*b* = −0.01, 95% CI −0.36 to 0.36, *P* = 0.94), or duration of treatment (*b* = −0.004, 95% CI −0.03 to 0.02, *P* = 0.63) and HbA_1c_. It was not possible to conduct sub‐analyses for interventionist category. It was not possible to perform meta‐regression for studies with an inclusion criterion of suboptimal glycaemic control as this included most of the studies in the meta‐analysis. See Tables S4 and S5, respectively for case definitions and whether HbA_1c_ was a primary or secondary outcome of included studies.

For children and adolescents, sub‐analyses for interventionist category demonstrated that there was a borderline statistically significant reduction in HbA_1c_ in favour of psychological interventions delivered by psychology professionals (*n* = 8, SMD −0.26, 95% CI −0.53 to 0.00, *P* = 0.05) equivalent to a reduction of 3 mmol/mol HbA_1c_ (0.3%), and a non‐significant reduction for ‘other’ interventionists (*n* = 7, SMD −0.02, 95% CI −0.12 to 0.08, *P* = 0.70), but a non‐significant increase for diabetes specialists (*n* = 5, SMD 0.03, 95% CI −0.34 to 0.40, *P* = 0.87). This difference was non‐significant in meta‐regression (*P* = 0.31). Heterogeneity was moderate and significant for psychology professionals (*I* ^2^ = 60.8%, *P* = 0.01), low and non‐significant for ‘other’ (*I* ^2^ = 0.0%, *P* = 0.92), and moderate and significant for diabetes specialist (*I* ^2^ = 73.7%, *P* = 0.004) studies. There was no association between number (*b* = −0.014, 95% CI −0.05 to 0.02, *P* = 0.40) or duration (*b* = −0.04, 95% CI −0.21 to 0.13, *P* = 0.60) of sessions, or duration of treatment (*b* = 0.0008, 95% CI −0.005 to 0.007, *P* = 0.79) and HbA_1c_. Meta‐regression revealed a non‐significant difference in HbA_1c_ between control group categories (*b* = −0.09, 95% CI −0.53 to 0.34, *P* = 0.63). Sub‐analyses for interventionist category demonstrated a non‐significant reduction in HbA_1c_ for counselling (*n* = 9, SMD −0.00, 95% CI −0.18 to 0.17, *P* = 0.99) and CBT (*n* = 7, SMD −0.24, 95% CI −0.58 to 0.09, *P* = 0.15). A meta‐regression found no significant difference between these group effect sizes (*P* = 0.46). A meta‐regression comparing studies that had an inclusion criterion of suboptimal glycaemic control (5 of 20) vs. those that did not demonstrated no difference between the two groups effect sizes (*b* = 0.25, 95% CI −0.11 to 0.60, *P* = 0.16). See Tables S6 and S7, respectively for case definitions and whether HbA_1c_ was a primary or secondary outcome of included studies.

### Network meta‐analysis synthesis

Network meta‐analysis was conducted for nine adult studies, which included two categories of psychological intervention and three control conditions. One study, Ismail *et al*. [S2], had two treatment arms (CBT, counselling), therefore 19 treatment and control arms were included, sample size *n* = 1219 (Table 1). CBT (*N* = 7) and counselling (*N* = 3) were the psychological intervention categories, usual care (*N* = 4), waiting list (*N* = 3) and attention control (*N* = 2) were the comparators (Table 1). With five conditions, 10 contrasts were possible and six were investigated (Fig. S5). Estimated direct and indirect effects between interventions did not differ significantly (Table S8).

**Table 1:** Number of studies and arms included in the network meta‐analyses for adults with type 1 diabetes

Arm	*N* (%)t	Arm	*N*
CBT	7 (36.8)	T	352
Counselling	3 (15.8)	T	299
Usual care	4 (21.1)	C	301
Attention control	2 (10.5)	C	103
Waiting list	3 (15.8)	C	164
Total	19 (100)		1219

CBT, cognitive behavioural therapy; T, defined as treatment arm in original study; C, defined as control group in original study.

Table 2 shows the results of consistency network meta‐analysis comparing treatments against usual care. Only CBT and attention control show significant reduction of HbA_1c_ outcome compared with usual care. Effect sizes were small (CBT) or medium (attention control). The non‐significant χ^2^ test for inconsistency (χ^2^ (3) = 0.30, *P* = 0.96, *I* ^2^ = 0) supports model consistency. Table S9 summarizes pairwise comparisons of treatment effect. The rankogram (Fig. S6) and SUCRA (Table 3) indicate that attention control has highest probability of being the best treatment, followed by CBT.

**Table 2:** Summary of treatment effects compared with treatment as usual assuming common heterogeneity estimate for all treatment design comparisons for adults with type 1 diabetes

Treatment	*b*	95% CI	se	*z*	P‐value
Usual care	0				
CBT	−0.256	(−0.452 to −0.059)	0.1	−2.55	0.011
Counselling	−0.122	(−0.316 to 0.071)	0.099	−1.24	0.22
Attention control	−0.456	(−0.797 to −0.115)	0.174	−2.62	0.009
Waiting list	−0.017	(−0.281 to 0.247)	0.135	−0.13	0.90

*b*, standardized mean difference using treatment as usual as control group. The formulas for Hedges’ *g* in White and Thomas 24 are used.

CBT, cognitive behavioural therapy.

**Table 3:** Mean rank and surface under the cumulative curve for adults with type 1 diabetes derived from ranking probabilities

	Mean rank	SUCRA	Order of treatment
Usual care	4.4	0.1	5
CBT	2.1	0.7	3
Counselling	3.2	0.5	2
Attention control	1.1	1	1
Waiting list	4.3	0.2	4

SUCRA, surface under the cumulative curve; CBT, cognitive behavioural therapy.

Network meta‐analysis was conducted for 19 child/adolescent studies; therefore 19 treatment and 19 control arms were included (sample size *n* = 2589; Table 4). CBT (*N* = 7) and counselling (*N* = 9) were the main categories of psychological intervention, usual care (*N* = 9) and attention control (*N* = 8) main controls. With six conditions, 15 contrasts were possible and seven were investigated (Fig. S7).

**Table 4:** Number of studies and arms included in the network meta‐analyses for children and adolescents with type 1 diabetes

Treatment	*N* (%)	Arm	*N*
CBT	7 (18.4)	T	266
Counselling	9 (23.7)	T	780
Usual care	9 (23.7)	C	970
Attention control	8 (21.1)	C	258
Family therapy	3 (7.9)	T	283
Waiting list	2 (5.3)	C	32
Total	38 (100)		2589

CBT, cognitive behavioural therapy; T, defined as treatment arm in original study; C, defined as control group in original study.

Estimated direct and indirect effects between interventions did not differ significantly (Table S10). Table 5 shows results of consistency network meta‐analysis comparing treatments against usual care. The non‐significant χ^2^ test for inconsistency (χ^2^ (2) = 0.90, *P* = 0.64, *I* ^2^ = 0) supports model consistency. No treatment showed a significant reduction of treatment outcome compared with usual care. Pairwise comparisons are shown in Table S11.

**Table 5:** Summary of treatment effects compared with treatment as usual assuming common heterogeneity estimate for all treatment design comparisons for children and adolescents with type 1 diabetes

Treatment	*b*	95% CI	se	*z*	*P*‐value
Usual care	0				
CBT	−0.332	(−1.204 to 0.541)	0.445	−0.75	0.46
Counselling	0.164	(−0.655 to 0.983)	0.418	0.39	0.69
Attention control	−0.267	(−1.239 to 0.706)	0.496	−0.54	0.59
Family therapy	−0.106	(−1.298 to 1.085)	0.608	−0.17	0.86
Waiting list	0.179	(−1.41 to 1.767)	0.81	0.22	0.83

*b*, standardized mean difference using treatment as usual as control group. The formulas for Hedges’ *g* in White and Thomas 24 are used.

CBT, cognitive behavioural therapy.

The rankogram (Fig. S8) and SUCRA (Table 6) indicate that CBT and attention control had the highest probability of being the best treatment.

**Table 6:** Mean rank and surface under the cumulative curve for children and adolescents with type 1 diabetes derived from ranking probabilities

Treatment	Mean rank	SUCRA	Order of treatment
Usual care	3.8	0.4	4
CBT	2.4	0.7	1
Counselling	4.4	0.3	6
Attention control	2.7	0.7	1
Family therapy	3.4	0.5	3
Waiting list	4.2	0.4	4

SUCRA, surface under the cumulative curve; CBT, cognitive behavioural therapy.

## Discussion

We conducted a systematic review and meta‐analysis of psychological interventions for children/adolescents and adults with type 1 diabetes. We identified 48 studies, 29 of which had HbA_1c_ data that could be included in a meta‐analysis. The main findings indicate that, in contrast to our earlier study 14, psychological interventions were not more effective than control conditions in improving glycaemic control for children/adolescents with type 1 diabetes. In accordance with the earlier review, glycaemic control was not improved in adults. The negative findings did not change when data from the earlier review were pooled. Therefore, the findings from the current review are likely to be more reliable considering that trial reporting standards have improved and so has the quality of the included studies 25. For both children/adolescent and adult studies, there was no evidence of a dose–response relationship in terms of intensity of psychological treatment, although most interventions involved fewer than eight sessions. For children and adolescents, there was a trend indicating that interventions delivered by psychology professionals were associated with a small improvement/reduction in HbA_1c_ of ~ 3mmol/mol (0.3%), although not significantly different from interventions delivered by diabetes specialists or other interventionists.

We were able to conduct network meta‐analysis and for adults, taking all comparisons into account, attention control and CBT interventions were associated with significant improvement/reduction in HbA_1c_ compared with usual care; attention control having the largest effect size and probability of being the best treatment. One explanation for this is that attention control groups involved interventions specific to diabetes self‐management, including blood glucose awareness training 26 and diabetes education 27. Therefore, it is possible that in addition to attention control interventions, CBT for adults with type 1 diabetes has potential to support and improve diabetes self‐management 28 and HbA_1c_.

Strengths of this study are that it was protocolized, registered with PROSPERO and conducted according to PRISMA guidelines 18. We attempted to identify published and grey literature, conducted hand‐searching and our search was not language restricted. Authors were contacted for missing data and we used two meta‐analytic approaches to synthesize data.

Limitations are that we were unable to determine from included studies whether participants had prior access to psychological therapy that may have influenced response to treatment, nor was it possible to determine whether therapists were competent to deliver the psychological treatment. However, the majority of adult studies were either manualized or provided a link to the study protocol, whereas few child/adolescent studies did. It could be argued that some of the psychological therapies such as motivational interviewing which is therapist‐led and was included under the counselling umbrella, are not as person‐centred as traditional counselling therapy. However, keeping MI within this category allowed us to merge data with our previous review 14 and there were too few studies overall to conduct meta‐regression. Despite having no language restriction, we identified most studies from North America and Western Europe. This review focused on glycaemic control and did not detect other potential benefits of psychological treatments, such as emotional well‐being and diabetes self‐management behaviour. These outcomes were considered in a separate report 29 although there was insufficient data that could be pooled in meta‐analysis. Also, this report focused on the nearest follow‐up to 12 months, and we acknowledge that although most studies included this time point there was variation and for some follow‐up was measured from baseline and for others post treatment. Furthermore, we also accept that reviewing studies with longer‐term interventions and follow‐up may be required.

Explanations as to why this systematic review and meta‐analysis demonstrated no effect of psychological treatments on glycaemic control for adults may be a consequence of the limited number of studies identified and the fact that the main outcome may not have been glycaemic control. Or for example, for some studies reduction in HbA_1c_ may not have been the main aim of psychological treatment if participants had hypoglycaemia unawareness 30. The reasons for the decreased effectiveness for psychological interventions for children and adolescents with type 1 diabetes are not clear and require international debate among diabetes and mental health professionals working in this field. For adults with type 1 diabetes, psychological interventions are predominantly limited to CBT and suggest that researchers may find it difficult to obtain funding for psychotherapies that are not on some government lists of approved therapies 31. For children/adolescents, there were a few large studies in which participants received a low dose of intervention and this appears too little to reverse the suboptimal glycaemic control which has existed for more than 10 years in the UK 32, and other developed countries 33. Treatment fidelity was not reported in any of the included studies and yet this is likely to influence the ‘dose’ of psychological treatment that is received 34.

Despite clinical guidelines suggesting the time of diagnosis an important time to offer psychological support 6, 7, 8, only one study specifically targeted children/adolescents at this time 35. Future studies may want to clearly define the target group and underlying issue being addressed, focus on therapies that potentially offer benefit such as CBT for adults, look to combine psychological and self‐management support, and look to develop targeted novel psychotherapies.

Therefore, although guidelines for the treatment of children and adults with type 1 diabetes recommend access to psychological services, there is no evidence to suggest that psychological treatments overall improve glycaemic control.

## Funding sources

This paper presents independent research funded by the UK's National Institute for Health Research (NIHR) Health Technology Assessment (HTA) Evidence Synthesis Programme (reference: 12/213/10). The views expressed are those of the authors and not necessarily those of the National Health Service (NHS), the NIHR or the Department of Health and Social Care.

The study is registered with PROSPERO, CRD42016033619.

## Competing interests

KW has served as a consultant or speaker for MSD and Valotech, SRH has served as a consultant for Lilly, Novo Nordisk, Takeda, Boeringher Ingelheim, Mannkind, Sanofi, Zealand Pharma and UN‐EEG. He is a recipient of an award from the NIHR to evaluate a complex intervention, DAFNEplus, designed to improve glycaemic control in adults with Type 1 diabetes. KI has received honorarium for educational lectures for Jannssen, Sanofi, Eli Lilly and Novo Nordisk.

## Author contributions

KW and KI conceived the study and DS, SH and AB made substantial contributions to the study design. RU conducted the literature search. KW and RU acquired the study data. RU, DS and DP conducted data analysis. KW, RU, DS and DP interpreted the data. RU and DS produced the figures. KW wrote the manuscript with substantial contributions, critical review and revision of the manuscript from RU, DS and KI. DP, SH and AB provided critical review and revision of the manuscript. All authors provided final approval for the publication of the manuscript. All authors agreed to be accountable for all aspects of the work in ensuring that questions related to the accuracy or integrity of the work are appropriately investigated and resolved.

## Supporting information


**Doc. S1.** Supplementary references.
**Table S1.** Search strategy for the systematic review of psychological interventions for people with type 1 diabetes.
**Table S2.** Summary of study characteristics for adults with type 1 diabetes.
**Table S3.** Summary of study characteristics for children and adolescents with type 1 diabetes.
**Table S4.** Case definition of type 1 diabetes adult studies included in meta‐analysis.
**Table S5.** Primary outcome of type 1 diabetes adult studies included in the meta‐analysis.
**Table S6.** Case definition of type 1 diabetes children/adolescent studies included in the meta‐analysis.
**Table S7.** Primary outcome of type 1 diabetes child and adolescent studies included in meta‐analysis.
**Table S8.** Direct and indirect treatment effects (where indirect treatment effects were available) and the difference between them, including significance test for difference in studies for adults with type 1 diabetes.
**Table S9.** Summary of pairwise comparisons of all treatment assuming common heterogeneity estimate for all treatment design comparisons for adults with type 1 diabetes. SMD=Standardised mean difference.
**Table S10.** Direct and indirect treatment effects (where indirect treatment effects were available) and the difference between them, including standard errors (SE) and significance test for difference in studies for children and adolescents with type 1 diabetes.
**Table S11.** Summary of all pairwise comparisons of treatment effects assuming common heterogeneity estimate for all treatment design comparisons for children with type 1 diabetes. SMD=standardised mean difference.
**Figure S1.** Risk of bias domain assessment across studies for adults with type 1 diabetes.
**Figure S2.** Risk of bias within studies for adults with type 1 diabetes.
**Figure S3.** Risk of bias domain assessment across studies for children/adolescents with type 1 diabetes.
**Figure S4.** Risk of bias within studies for children/adolescents with type 1 diabetes.
**Figure S5.** Network plots of direct comparisons for the network meta‐analysis for adults with type 1 diabetes.
**Figure S6.** Rankogram for all treatments for adults with type 1 diabetes.
**Figure S7.** Network plots of direct comparisons for the network meta‐analysis for children and adolescents with type 1 diabetes.
**Figure S8.** Rankogram for all treatments for children and adolescents with type 1 diabetes.
