# Supplementary material for: Systematic review and meta‐analysis of randomized controlled trials of psychological interventions to improve glycaemic control in children and adults with type 1 diabetes
Source: Diabet Med. 2020 Mar 10;37(5):735–46. doi: 10.1111/dme.14264 (PMC7217004; doi:10.1111/dme.14264)
Supplement: Supplementary file 1 — Doc. S1. Supplementary references. Table S1. Search strategy for the systematic review of psychological interventions for people with type 1 diabetes. Table S2. Summary of study characteristics for adults with type 1 diabetes. Table S3. Summary of study characteristics for children and adolescents with type 1 diabetes. Table S4. Case definition of type 1 diabetes adult studies included in meta‐analysis. Table S5. Primary outcome of type 1 diabetes adult studies included in the meta‐analysis. Table S6. Case definition of type 1 diabetes children/adolescent studies included in the meta‐analysis. Table S7. Primary outcome of type 1 diabetes child and adolescent studies included in meta‐analysis. Table S8. Direct and indirect treatment effects (where indirect treatment effects were available) and the difference between them, including significance test for difference in studies for adults with type 1 diabetes. Table S9. Summary of pairwise comparisons of all treatment assuming common heterogeneity estimate for all treatment design comparisons for adults with type 1 diabetes. SMD=Standardised mean difference. Table S10. Direct and indirect treatment effects (where indirect treatment effects were available) and the difference between them, including standard errors (SE) and significance test for difference in studies for children and adolescents with type 1 diabetes. Table S11. Summary of all pairwise comparisons of treatment effects assuming common heterogeneity estimate for all treatment design comparisons for children with type 1 diabetes. SMD=standardised mean difference. Figure S1. Risk of bias domain assessment across studies for adults with type 1 diabetes. Figure S2. Risk of bias within studies for adults with type 1 diabetes. Figure S3. Risk of bias domain assessment across studies for children/adolescents with type 1 diabetes. Figure S4. Risk of bias within studies for children/adolescents with type 1 diabetes. Figure S5. Network plots of direct comparisons for [file DME-37-735-s001.docx]

**Systematic review and meta-analysis of randomized controlled trials of psychological interventions to improve glycaemic control in children and adults with type 1 diabetes**

K. Winkley, R. Upsher, D. Stahl, D. Pollard, A. Brennan, S. Heller and K. Ismail

**Doc. S1. Supplementary references**

S1. Amsberg S, Anderbro T, Wredling R, Lisspers J, Lins PE, Adamson U, et al. A cognitive behavior therapy-based intervention among poorly controlled adult type 1 diabetes patients-A randomized controlled trial. Patient Education and Counseling. 2009;77(1):72-80.

S2. Ismail K, Thomas SM, Maissi E, Chalder T, Schmidt U, Bartlett J, et al. Motivational enhancement therapy with and without cognitive behavior therapy to treat type 1 diabetes: A randomized trial. Annals of Internal Medicine. 2008;149(10):708-19.

S3. Menting J, Tack CJ, van Bon AC, Jansen HJ, van den Bergh JP, Mol MJTM, et al. Web-based cognitive behavioural therapy blended with face-to-face sessions for chronic fatigue in type 1 diabetes: a multicentre randomised controlled trial. The Lancet Diabetes and Endocrinology. 2017;5(6):448-56.

S4. Mohn J, Graue M, Assmus J, Zoffmann V, Thordarson H, Peyrot M, et al. The effect of guided self-determination on self-management in persons with type 1 diabetes mellitus and HbA 1c >=64 mmol/mol: A group-based randomised controlled trial. BMJ Open. 2017;7(6):e013295.

S5. Pyatak E, Carandang K, Vigen C, Blanchard J, Diaz J, Concha-Chavez A, et al. Occupational therapy intervention improves glycemic control and quality of life among young adults with diabetes: the resilient, empowered, active living with diabetes (REAL Diabetes) randomized controlled trial. Diabetes care. 2018;41(4):696-704.

S6. Snoek FJ, Van Der Ven NCW, Twisk JWR, Hogenelst MHE, Tromp-Wever AME, Van Der Ploeg HM, et al. Cognitive behavioural therapy (CBT) compared with blood glucose awareness training (BGAT) in poorly controlled Type 1 diabetic patients: Long-term effects on HbA1c moderated by depression. A randomized controlled trial. Diabetic Medicine. 2008;25(11):1337-42.

S7. Tovote K, Schroevers M, Snippe E, Emmelkamp P, Links T, Sanderman R, et al. What works best for whom? Cognitive Behavior Therapy and Mindfulness-Based Cognitive Therapy for depressive symptoms in patients with diabetes. Plos one. 2017;12(6):e0179941.

S8. Weinger K, Beverly EA, Lee Y, Sitnokov L, Ganda OP, Caballero AE. The effect of a structured behavioral intervention on poorly controlled diabetes: A randomized controlled trial. Diabetes Technology and Therapeutics. 2013;15(SUPPL.1):S9.

S9. Zoffmann V, Vistisen D, Due-Christensen M. Flexible guided self-determination intervention for younger adults with poorly controlled Type 1 diabetes, decreased HbA1c and psychosocial distress in women but not in men: a real-life RCT. Diabetic Medicine. 2015;32(9):1239-46.

S10. Fischer HH, Eisert SL, Everhart RM, Durfee MJ, Moore SL, Soria S, et al. Nurse-run, telephone-based outreach to improve lipids in people with diabetes. The American journal of managed care. 2012;18(2):77-84.

S11. Friis A, Johnson M, Cutfield R, Consedine N. Kindness Matters: a Randomized Controlled Trial of a Mindful Self-Compassion Intervention Improves Depression, Distress, and HbA1c Among Patients With Diabetes. Diabetes care. 2016;39(11):1963-71.

S12. Hermanns N, Schmitt A, Reimer A, Ehrmann D, Haak T, Kulzer B. The Effect of a Diabetes-Specific Cognitive Behavioral Treatment Program (DIAMOS) for Patients with Diabetes and Subclinical Depression: Results of a Randomized Controlled Trial. Diabetes Care 2015;38:551-560. Diabetes Care. 2016;39(1):e13-e4.

S13. Karlsen B, Idsoe T, Dirdal I, Hanestad BR, Bru E. Effects of a group-based counselling programme on diabetes-related stress, coping, psychological well-being and metabolic control in adults with type 1 or type 2 diabetes. Patient education and counseling. 2004;53(3):299-308.

S14. Katon WJ, Von Korff M, Lin EH, Simon G, Ludman E, Russo J, et al. The pathways study: A randomized trial of collaborative care in patients with diabetesand depression. Archives of general psychiatry. 2004;61(10):1042-9.

S15. Minet LK, Wagner L, Lonvig EM, Hjelmborg J, Henriksen JE. The effect of motivational interviewing on glycaemic control and perceived competence of diabetes self-management in patients with type 1 and type 2 diabetes mellitus after attending a group education programme: A randomised controlled trial. Diabetologia. 2011;54(7):1620-9.

S16. Petrak F, Herpertz S, Albus C, Hermanns N, Hiemke C, Hiller W, et al. Cognitive behavioral therapy versus sertraline in patients with depression and poorly controlled diabetes: The Diabetes and Depression (DAD) Study: A randomized controlled multicenter trial. Diabetes Care. 2015;38(5):767-75.

S17. Rondags S, de Wit M, Twisk JW, Snoek FJ. Effectiveness of HypoAware, a Brief Partly Web-Based Psychoeducational Intervention for Adults With Type 1 and Insulin-Treated Type 2 Diabetes and Problematic Hypoglycemia: A Cluster Randomized Controlled Trial. Diabetes Care. 2016;39(12):2190-6.

S18. Safford MM, Andreae S, Cherrington AL, Martin MY, Halanych J, Lewis M, et al. Peer coaches to improve diabetes outcomes in rural Alabama: a cluster randomized trial. The Annals of Family Medicine. 2015;13(Suppl 1):S18-S26.

S19. Schroevers MJ, Tovote KA, Keers JC, Links TP, Sanderman R, Fleer J. Individual mindfulness-based cognitive therapy for people with diabetes: a pilot randomized controlled trial. Mindfulness. 2015;6(1):99-110.

S20. van Son J, Nyklicek I, Pop VJ, Blonk MC, Erdtsieck RJ, Pouwer F. Mindfulness-based cognitive therapy for people with diabetes and emotional problems: Long-term follow-up findings from the DiaMind randomized controlled trial. Journal of Psychosomatic Research. 2014;77(1):81-4.

S21. Williams ED, Bird D, Forbes AW, Russell A, Ash S, Friedman R, et al. Randomised controlled trial of an automated, interactive telephone intervention (TLC Diabetes) to improve type 2 diabetes management: baseline findings and six-month outcomes. BMC public health. 2012;12(1):602.

22. Zoffmann V, Lauritzen T. Guided self-determination improves life skills with Type 1 diabetes and A1C in randomized controlled trial. Patient Education and Counseling. 2006;64(1-3):78-86.

S23. Heisler M, Vijan S, Makki F, Piette JD. Diabetes control with reciprocal peer support versus nurse care management: a randomized trial. Annals of internal medicine. 2010;153(8):507.

S24. Lin EH, Von Korff M, Peterson D, Ludman EJ, Ciechanowski P, Katon W. Population targeting and durability of multimorbidity collaborative care management. The American journal of managed care. 2014;20(11):887.

S25. Channon SJ, Huws-Thomas MV, Rollnick S, Hood K, Cannings-John RL, Rogers C, et al. A multicenter randomized controlled trial of motivational interviewing in teenagers with diabetes. Diabetes care. 2007;30(6):1390-5.

S26. Christie D, Thompson R, Sawtell M, Allen E, Cairns J, Smith F, et al. Structured, intensive education maximising engagement, motivation and long-term change for children and young people with diabetes: a cluster randomised controlled trial with integral process and economic evaluation-the CASCADE study. Health Technology Assessment (Winchester, England). 2014;18(20):1.

S27. Ellis DA, Naar-King S, Chen X, Moltz K, Cunningham PB, Idalski-Carcone A. Multisystemic therapy compared to telephone support for youth with poorly controlled diabetes: Findings from a randomized controlled trial. Annals of Behavioral Medicine. 2012;44(2):207-15.

S28. Ellis DA, Templin T, Naar-King S, Frey MA, Cunningham PB, Podolski C-L, et al. Multisystemic therapy for adolescents with poorly controlled type I diabetes: Stability of treatment effects in a randomized controlled trial. Journal of consulting and clinical psychology. 2007;75(1):168.

S29. Grey M, Whittemore R, Jaser S, Ambrosino J, Lindemann E, Liberti L, et al. Effects of coping skills training in school‐age children with type 1 diabetes. Research in nursing & health. 2009;32(4):405-18.

S30. Harris MA, Freeman KA, Duke DC. Seeing is believing: using Skype to improve diabetes outcomes in youth. Diabetes Care. 2015;38(8):1427-34.

S31. Holmes CS, Chen R, Mackey E, Grey M, Streisand R. Randomized clinical trial of clinic-integrated, low-intensity treatment to prevent deterioration of disease care in adolescents with type 1 diabetes. Diabetes Care. 2014;37(6):1535-43.

S32. Husted GR, Thorsteinsson B, Esbensen BA, Gluud C, Winkel P, Hommel E, et al. Effect of guided self-determination youth intervention integrated into outpatient visits versus treatment as usual on glycemic control and life skills: a randomized clinical trial in adolescents with type 1 diabetes. Trials. 2014;15(1):321.

S33. Jaser SS, Patel N, Linsky R, Whittemore R. A Randomized Pilot of a Positive Psychology Intervention to Improve Adherence in Adolescents With Type 1 Diabetes. Journal of Pediatric Health Care. 2014;28(6):478-85.

S34. Katz ML, Volkening LK, Butler DA, Anderson BJ, Laffel LM. Family‐based psychoeducation and care ambassador intervention to improve glycemic control in youth with type 1 diabetes: a randomized trial. Pediatric diabetes. 2014;15(2):142-50.

S35. Lehmkuhl HD, Storch EA, Cammarata C, Meyer K, Rahman O, Silverstein J, et al. Telehealth behavior therapy for the management of type 1 diabetes in adolescents. Journal of diabetes Science and Technology. 2010;4(1):199-208.

S36. Mackey ER, Herbert L, Monaghan M, Cogen F, Wang J, Streisand R. The feasibility of a pilot intervention for parents of young children newly diagnosed with type 1 diabetes. Clinical Practice in Pediatric Psychology. 2016;4(1):35-50.

S37. Najmi SB, Marasi MR, Hashemipour M, Hovsepian S, Ghasemi M. The perceived self-efficacy and its interrelation with communication in family and glycemic control in adolescents with type 1 diabetes. Pakistan Journal of Medical Sciences. 2013;29(1 SUPPL.):334-9.

S38. Nansel TR, Iannotti RJ, Liu A. Clinic-integrated behavioral intervention for families of youth with type 1 diabetes: randomized clinical trial. Pediatrics. 2012;129(4):e866-e73.

S39. Nansel TR, Iannotti RJ, Simons-Morton BG, Cox C, Plotnick LP, Clark LM, et al. Diabetes personal trainer outcomes: short-term and 1-year outcomes of a diabetes personal trainer intervention among youth with type 1 diabetes. Diabetes Care. 2007;30(10):2471-7.

S40. Nansel TR, Laffel LM, Haynie DL, Mehta SN, Lipsky LM, Volkening LK, et al. Improving dietary quality in youth with type 1 diabetes: randomized clinical trial of a family-based behavioral intervention. International Journal of Behavioral Nutrition and Physical Activity. 2015;12(1):58.

S41. Robling M, McNamara R, Bennert K, Butler CC, Channon S, Cohen D, et al. The effect of the Talking Diabetes consulting skills intervention on glycaemic control and quality of life in children with type 1 diabetes: cluster randomised controlled trial (DEPICTED study). Bmj. 2012;344:e2359.

S42. Wang Y-C, Stewart SM, Mackenzie M, Nakonezny PA, Edwards D, White PC. A randomized controlled trial comparing motivational interviewing in education to structured diabetes education in teens with type 1 diabetes. Diabetes care. 2010;33(8):1741-3.

S43. Wei C, Allen RJ, Tallis PM, Ryan FJ, Hunt LP, Shield JPH, et al. Cognitive behavioural therapy stabilises glycaemic control in adolescents with type 1 diabetes-Outcomes from a randomised control trial. Pediatric Diabetes. 2018;19(1):106-13.

S44. Wysocki T, Harris MA, Buckloh LM, Mertlich D, Lochrie AS, Taylor A, et al. Effects of behavioral family systems therapy for diabetes on adolescents’ family relationships, treatment adherence, and metabolic control. Journal of pediatric psychology. 2006;31(9):928-38.

S45. Graue M, Wentzel‐Larsen T, Hanestad B, Søvik O. Evaluation of a programme of group visits and computer‐assisted consultations in the treatment of adolescents with Type 1 diabetes. Diabetic Medicine. 2005;22(11):1522-9.

S46. Saßmann H, de Hair M, Danne T, Lange K. Reducing stress and supporting positive relations in families of young children with type 1 diabetes: A randomized controlled study for evaluating the effects of the DELFIN parenting program. BMC pediatrics. 2012;12(1):152.

S47. Serlachius A, Scratch S, Northam E, Frydenberg E, Lee K, Cameron F. A randomized controlled trial of cognitive behaviour therapy to improve glycaemic control and psychosocial wellbeing in adolescents with type 1 diabetes. Journal of health psychology. 2016;21(6):1157-69.

**Table S1. Search strategy for the systematic review of psychological interventions for people with type 1 diabetes**

| **Keyword** | **Diabetes mellitus** |  | **Psychological therapies and Mood disorders** |  | **Clinical trials** |
| --- | --- | --- | --- | --- | --- |
| 1 | exp Diabetes Mellitus/ | - 21 | exp Psychotherapy/ | - 61 | Randomized Controlled Trials as Topic/ |
| 2 | diabet$.ab,ti. | - 22 | exp Counseling/ | - 62 | randomized controlled trial/ |
| 3 | (DKA or IDDM).mp. or DMI.ab,ti. [mp=title, original title, abstract, name of substance word, subject heading word] | - 23 | exp Mood disorders/ | 63 | Random Allocation/ |
| 4 | (MODY or DM2 or NIDDM).mp. or IIDM.ti,ab. [mp=title, original title, abstract, name of substance word, subject heading word] | - 24 | exp Depression/ | 64 | Double Blind Method/ |
| 5 | insulin$ secret$ dysfunc$.ti,ab. | - 25 | psycho$.mp | 65 | Single Blind Method/ |
| 6 | insulin$ resist$.ti,ab. | - 26 | counsel$.mp | 66 | clinical trial/ |
| 7 | ((impaired glucose tolerance or glucose intoleran$ or insulin$ resist$) and (DM or DM2)).ti,ab. | - 27 | depression.mp | 67 | clinical trial, phase i.pt |
| 8 | insulin$ depend$.mp. or insulin?depend$.ti,ab. [mp=title, original title, abstract, name of substance word, subject heading word] | - 28 | depressive.mp | 68 | clinical trial, phase ii.pt |
| 9 | (non insulin$ depend$ or nonisulin$ depend$ or nonisulin?depend).mp. or non insulin?depend$.ti,ab. [mp=title, original title, abstract, name of substance word, subject heading word] | - 29 | (interpersonal adj5 therap$).mp | 69 | clinical trial, phase iii.pt |
| 10 | (("typ$ 1" or typ$ I) adj6 DM).ti,ab. | - 30 | art therap$.mp | 70 | clinical trial, phase iv.pt |
| 11 | (("typ$ 2" or typ$ II) adj6 DM).ti,ab. | - 31 | aversion therap$.mp | 71 | controlled clinical trial.pt |
| 12 | ((juvenil$ or child$ or keto$ or labil$ or brittl$ or earl$ onset) adj6 (DM or DM1)).ti,ab. | - 32 | balint.mp | 72 | randomized controlled trial.pt |
| 13 | ((keto$ prone or autoimmun$ or auto immun$ or sudden onset) adj6 (DM or DM1)).ti,ab. | - 33 | behavio?r adj5 (intervention or therap* or modific*) | 73 | multicenter study.pt |
| 14 | ((keto$ resist$ or nonketo$ or non keto$ or adult$ onset or matur$ onset or late$ onset or slow onset or stabl$) adj6 (DM or DM2)).ti,ab. | - 34 | cognitive adj5 (therap* or intervention or program* or train* or theory) | 74 | clinical trial.pt |
| 15 | exp Insulin Resistance/ | - 35 | (family adj3 (intervention or treatment or counsel* or therap*) | 75 | exp Clinical Trials as topic/ |
| 16 | (insulin$ defic$ adj6 (absolut$ or relativ$)).ti,ab. | - 36 | colo?r therap$.mp. | 76 | (clinical adj25 trial$).tw |
| 17 | metabolic$ syndrom$.ti,ab. | - 37 | crisis intervention.mp | 77 | ((singl$ or doubl$ or treb$ or tripl$) adj25 (blind$3 or mask$3)).tw |
| 18 | (syndrom$ X not (fragil$ X or X linked)).ti,ab. | - 38 | dance therap$.mp | 78 | PLACEBOS/ |
| 19 | (plurimetabolic$ syndrom$ or pluri metabolic$ syndrom$).ti,ab. | - 39 | gestalt therap$.mp | 79 | placebo$.tw |
| 20 | or/1-19 | - 40 | music therap$.mp | 80 | randomly allocated.tw |
|  |  | - 41 | milieu therap$.mp | 81 | (allocated adj2 random$).tw |
|  |  | - 42 | (assert$ adj5 training).mp | 82 | Or/61-81 |
|  |  | - 43 | Narrative therap$.mp. | 83 | case report.tw |
|  |  | - 44 | nondirective therap$.mp | 84 | letter/ |
|  |  | - 45 | (problem solving adj5 therap$).mp | 85 | historical article/ |
|  |  | - 46 | (self control adj5 therap$).mp | 86 | Or/ 83-85 |
|  |  | - 47 | person cent$.mp | 87 | 82 NOT 86 |
|  |  | 48 | client cent$.mp | 88 | 20 AND 60 AND 87 |
|  |  | - 49 | psychodrama$.mp | 89 | limit 88 to yr="2003 -Current" |
|  |  | - 50 | paradoxical technique$.mp |  |  |
|  |  | 51 | play therap$.mp |  |  |
|  |  | - 52 | rational emotive.mp |  |  |
|  |  | - 53 | reality therap$.mp |  |  |
|  |  | - 54 | role play$.mp |  |  |
|  |  | - 55 | (relax$ adj5 training).mp |  |  |
|  |  | - 56 | sociotherap$.mp |  |  |
|  |  | - 57 | socioenvironmental.mp |  |  |
|  |  | - 58 | supportive therap$.mp |  |  |
|  |  | - 59 | transactional.mp |  |  |
|  |  | - 60 | acceptance adj2 (commitment therap*) |  |  |
|  |  | - 61 | coping skills training.mp. |  |  |
|  |  | - 62 | exp Mindfulness/ |  |  |
|  |  | - 63 | motivation* adj2 (interview* or therap*) |  |  |
|  |  | - 64 | multisystemic therapy |  |  |
|  |  |  |  |  |  |

**Table S2**. Summary of study characteristics for adults with type 1 diabetes

| **First author, year, country of study** | **N** | **Mean age (SD), years** | **Inclusion criteria of individual studies** | **Mean duration of diabetes (SD) in years** | **Number of sessions in intervention** | **Psychological intervention; intervention category; format; mode; interventionist** | **Control group; control category** |
| --- | --- | --- | --- | --- | --- | --- | --- |
| **Studies included in systematic review and meta-analysis** | | | | | | | |
| Amsberg, 2009, Sweden (S1) | 74 | All: 41.2 (12.3) | Diabetes duration: ≥2 years  Age: 18-65 years  HbA1c: >58 mmol/mol, 7.5% | All: 21.6 (10.8) | 8 | CBT; CBT; face to face; group; diabetes specialist nurse and psychologist | Waiting list; Usual care |
| Ismail, 2008, UK (S2) | 344 | All: 58.9 (11.2) | Diabetes duration: ≥2 years  Age: 18-65 years  HbA1c: 66-21 mmol/mol, 8.2-15% | All: 9 (6-12, IQR) | 12 | MET+CBT; CBT; face to face; individual; diabetes nurse | 1. Usual care 2. MET; Counselling |
| Snoek, 2008, Netherlands (S6) | 86 | I: 38.1 (9.7)  C: 37.4 (11.1) | Diabetes duration: ≥1 year  HbA1c: ≥64mmol/mol, 8% | I: 17.8 (10.1)  C: 18.8 (10.9) | 6 | Cognitive behavioural group training; CBT; face to face; group; psychologist | BGAT; Attention control |
| Hermanns, 2015, Germany (S12) | 114 | I: 43.2 (14.9)  C: 43.4 (13.8) | Age: ≥18 & ≤70 years  Other: Depressive symptoms | I: 14.2 (10.3)  C: 14.2 (10.7) | 5 | CBT-based; CBT; face to face; group; psychologist | Diabetes education; attention control |
| Zoffmann, 2015, Denmark (S9) | 200 | I: 25.9 (5.0)  C:25.3 (5.2) | Diabetes duration: ≥1 year  Age: 18-35 years  HbA1c: ≥ 64 mmol/mol, 8% | I: 13.8 (6.9)  C: 13.7 (6.8) | 14 | GSD; counselling; face-to-face; group; diabetes nurse | Waiting list |
| Van Son, 2014, Netherlands (S20) | 83 | I: 56 (13)  C:57 (13) | Age: ≥18 years  Other: Low emotional well-being | Not reported | 8 | MBCT; CBT; face to face; group; psychologist | Usual care |
| Petrak, 2015, Germany (S16) | 53 | All: 48.5 (11.7) | Age: 21-69 years  HbA1c: >58mmol/mol, 7.5%  Other: Depressive symptoms | All: 15.3 (10.5) | 10 | CBT; CBT; face-to-face; group; psychologist | Usual care |
| Mohn, 2017, Norway (S4) | 131 | I: 36.9 (9.4)  C:37.2 (10.9) | Diabetes duration: ≥1 year  Age: 18-55 years  HbA1c: ≥64 mmol/mol, 8% | I: 18.5 (10.6)  C: 20.6 (11.2) | 7 | GSD; counselling; face-to-face; group; diabetes nurse | Usual care |
| Menting, 2017, Netherlands (S3) | 120 | I: 44.4 (12.1)  C: 42.9 (12.5) | Diabetes duration: ≥1 year  Age: 18-70 years | I: 24.2 (13.3)  C: 24.1 (13.9) | 6.5 | CBT; CBT; face to face; individual; therapist | Waiting list |
| **Studies included in systematic review only** | | | | | | | |
| Zoffmann, 2006, Denmark (S22) | 50 | I: 36.8 (1.7)  C: 25.7 (2.1) | Age: 18-49 years  HbA1c: ≥64mmol/mol, 8% | Not reported | 8 | GSD; counselling; face-to-face; group; diabetes nurse | Waiting list |
| Weinger, 2011, USA (S8) | 222 | I: 51.8 (23.7-74.2)  C1: 54.7 (25-75.1)  C2: 56.2 (21.6-74.8)  [median, range] | Diabetes duration: ≥2 years  Age: 18-70 years  HbA1c: >58 mmol/mol, 7.5% | I: 14.9 (1.3-66.1)  C1: 15.0 (2.6-48.5)  C2: 16.8 (2.2-45.7) | 5 | Structured behavioural group; CBT; face to face, group; diabetes educators | 1. Group attention control, attention control 2. Individual control; attention control |
| Karlsen, 2004, Norway (S13) | 63 | I: 49.2 (14.7)  C:48.6 (10.3) | Age: 26-70 years | Not reported | 9 | Group-based counselling; CBT; face to face; group; diabetes nurse | Waiting list |
| Minet, 2011, Denmark (S15) | 349 | I: 57.1 (12.6)  C: 55.8 (11.6) | Age: >18 years | I: 4.7 (6.9)  C:4.7 (6.5) | 5 | MI; counselling; face to face; individual; HCPs (nurse, dietician, physiotherapist or psychologist) | Usual care |
| Schroevers, 2015, Netherlands (S19) | 24 | I: 54.9 (10.3)  C:55.9 (8.2) | Age: 18-70 years | I: 16.6 (14.4)  C:20.5 (13.7) | 8 | MBCT; counselling; face to face; individual; psychologist | Waiting list |
| Safford, 2015, USA (S18) | Not reported per diabetes type | All: 60.2 (12.1) | Age: ≥18 years | Not reported | Variable | MI; counselling; telephone; individual; peers | Diabetes education; attention control |
| Lin, 2014, USA (S24) | Not reported per diabetes type | I: 57.4 (10.5)  C: 56.3 (12.1) | Other: Depressive symptoms | Not reported | Variable | Collaborative care; counselling; face to face; individual; primary care physician & nurse & psychiatrist & psychologist | Usual care |
| Katon, 2004, USA (S14) | 329 | I: 58.6 (11.8)  C: 58.1 (12.0) | Age: ≥18 years | I: 9.6 (8.8)  C: 10.2 (10.1) | Variable | Pathways study; CBT (problem-solving treatment); face-to-face/telephone; individual; nurses/psychiatrists/primary care physician | Usual care |
| Heisler, 2010, Germany (S23) | 244 | All: 62.0 (6.3) | HbA1c: >58mmol/mol, 7.5% | Not reported | Variable | Nurse case management; counselling; face-to-face and telephone; individual; diabetes nurse | Reciprocal Peer Support; peer support |
| Williams, 2012, Australia (S21) | 80 | I: 68 (8.3)  C: 66 (10.8) | Age: ≥18 years | Not reported | Variable | Self-management intervention; counselling; face-to-face and telephone; individual; diabetes nurse | Usual care |
| Fischer, 2012, USA (S10) | 762 | I: 58.5 (12.4)  C: 58.3 (12.1) | Age: >17 years | Not reported | Variable | Telephone-Based Outreach; counselling; telephone; individual; diabetes nurse | Usual care |
| Friis, 2016, New Zeland (S11) | 46 | All: 44.37 (15.62) | Age: 18-70 years | All: 16.84 (12.32) | 8 | Mindful self-compassion program, counselling; face to face; group; therapist | Waiting list |
| Pyatak, 2017, USA (S5) | 61 | All: 2.6 (3.5) | Diabetes duration: ≥1 year  Age: 18-30 years  HbA1c: ≥64 mmol/mol, 8% | All: 9.7 (5.8) | Variable | REAL Diabetes  intervention group; counselling; face to face; individual; therapist | Attention control; attention control |
| Rondags, 2016, Netherlands (S17) | 121 | I: 52.7 (12.4)  C: 51.3 (14.0) | Age: ≥18 years | I: 24.6 (14.0)  C:27.5 (13.1) | 3 | HypoAware; counselling; face to face; group; diabetes nurse | Usual care |
| Tovote, 2017, Finland (S7) | 35 | All: 53.2 (11.9) | Diabetes duration: ≥3 months  Age: 18-70 years  Other: depressive symptoms | All: 16.7 (12.0) | 8 | CBT; CBT; face to face; individual; therapist | MBCT; attention control |

CBT= cognitive behavioural therapy; MET= motivational enhancement therapy; BGAT= blood glucose awareness training; GSD=guided self-determination therapy; MBCT=mindful-based cognitive therapy

**Table S3.** Summary of study characteristics for children and adolescents with type 1 diabetes

| **First author, year, country of study** | **N** | **Mean age (SD), years** | **Inclusion criteria of individual studies** | **Mean duration of diabetes (SD) in years** | **Number of sessions in intervention** | **Psychological intervention; intervention category; format; mode; interventionist** | **Control group; control category** |
| --- | --- | --- | --- | --- | --- | --- | --- |
| **Studies included in systematic review and meta-analysis** | | | | | | | |
| Graue, 2005, Norway (S45) | 83 | I: 14.5 (1.6)  C: 14.3 (1.6) | Age: 11-17 years | I: 6.7 (3.3)  C:6.9 (4.3) | 3 | Structured educational and counselling programme; counselling; face-to-face, group; physician/diabetes specialist nurse/ clinical psychologist/ dietician/ social worker | Usual care |
| Channon, 2007, UK (S25) | 66 | I: 15.3 (0.97)  C:15.4 (1.19) | Diabetes duration: ≥1 year  Age: 14-17 years | I: 9.2 (1.96)  C:9.1 (1.47) | 4 | MI; counselling; face to face; individual; diabetes nurse | nondirective  psychological support; attention control |
| Ellis, 2007, USA (S28) | 127 | All: 13.2 (1.9) | Diabetes duration: ≥1 year  Age: 11-16 years  HbA1c: ≥64 mmol/mol, 8% | Not reported | Not reported | MST; family systems therapy; fact to face; family; therapist | Usual care |
| Nansel, 2007, USA (S39) | 81 | All: 13.8 (1.7) | Diabetes duration: ≥1 year  Age: 11-16 years | All: 7.7 (3.7) | 6 | Diabetes personal trainer intervention; counselling; face to face/telephone; family/individual; MI-trained non-professional | Diabetes education; attention control |
| Grey, 2009, USA (S29) | 82 | I: 9.9 (1.5)  C: 9.9 (1.4) | Diabetes duration: ≥6 months  Age: 8-12 years | I: 3.7 (2.78)  C: 3.6 (3.0) | 6 | CST; CBT; face-to-face; group; mental health professional | Diabetes education; attention control |
| Wang, 2010, USA (S42) | 44 | I: 15.3 (1.4)  C: 15.6 (1.7) | Diabetes duration: ≥1 year  Age: 12-18 years  HbA1c: ≥ 75 mmol/mol, 9 % | I: 6.7 (3.4)  C: 7.6 (4.7) | 2 | MI; counselling; face-to-face; group; diabetes educator | Diabetes education; attention control |
| Lehmkuhl, 2010, USA (S35) | 32 | I: 13.72 (2.67)  C:13.43 (2.17) | Diabetes duration: ≥6 months  HbA1c: >75 mmol/mol, 9% | Not reported | 36 | Telehealth behaviour; family systems therapy; telephone; family; clinical psychologists or clinical psychology interns | Waiting list; usual care |
| Robling, 2012, UK (S41) | 689 | All: 10.6 (2.8) | Diabetes duration: ≥1 year  Age: 4-15 years | All: 5.1 (2.7) | Variable | DEPICTED; counselling; face to face; individual; healthcare professionals | Usual care |
| Sassmann, 2012, Germany (S46) | 33 | I: 6.4 (2.3)  C: 5.8 (1.9) | Age: 2-10 years | I: 2.6 (1.6)  C:2.6 (1.9) | 5 | DELFIN; CBT; face-to-face, group; psychologist | Waiting list; usual care |
| Nansel, 2012, USA (S38) | 390 | I: 12.5 (1.8)  C:12.4 (1.7) | Diabetes duration: ≥3 months  Age: 9-14.9 years  HbA1c: >42 mmol/mol, 6% | I: 4.8 (3.3)  C:4.9 (3.2) | 9 | Family behavioural intervention; family systems therapy; face to face/telephone; family; health advisors | Usual care |
| Najmi, 2012, Iran (S37) | 85 | All: 14.9 (1.8) | Diabetes duration: ≥1 year  Age: 12-18 years | Not reported | 8 | CBT+CST; CBT; face to face; family; psychiatrist | 1. Usual care 2. CBT; CBT 3. CST; counselling |
| Husted, 2014, Denmark (S32) | 71 | I: 14.9 (1.5)  C: 14.6 (1.3) | Diabetes duration: ≥1 year  Age: 13-18 years | I: 6.1 (3.0)  C:5.3 (3.4) | 8 | GSD; counselling; face to face; individual; physicians/diabetes nurses/dietician | Usual care |
| Jaser, 2014, USA (S33) | 40 | I: 15.3 (1.4)  C:15.0 (1.6) | Diabetes duration: ≥6 months  Age: 13-17 years | I: 7.3 (4.3)  C:6.5 (3.5) | 4 | Positive affect; counselling; telephone; family; trained research assistant | Diabetes education; attention control |
| Katz, 2014, USA (S34) | 153 | I: 12.7 (2.2)  C1: 13.4 (2.4)  C2: 12.5 (2.3) | Diabetes duration: ≥6 months  Age: 8-16 years | I: 6.5 (3.8)  C1:6.8 (3.2)  C2: 5.7 (3.5) | Variable | Care ambassador ultra; CBT; face to face; family; research assistant | 1. Usual care 2. Care ambassador via telephone; attention control |
| Christie, 2014, UK (S26) | 315 | I: 13.1 (2.1)  C: 13.2 (2.1) | Diabetes duration: ≥1 year  Age: 8-16 years  HbA1c: ≥68 mmol/mol, 8.5 % | I: 5.7 (3.2)  C: 6.1 (3.3) | 4 | CASCADE (psychoeducation); counselling; face to face; group; diabetes specialist nurse and another HCP | Usual care |
| Harris, 2015 (S30) | 90 | I: 15.04 (1.79)  C:14.94 (1.77) | Diabetes duration: ≥1 year  Age: 12-19 years  HbA1c: ≥75 mmol/mol, 9% | I: 6.51 (3.24)  C:6.56 (3.77) | 10 | BFST-D; family systems therapy; face to face; family; therapist | BFST-D via skype; attention control |
| Nansel, 2015 (S40) | 136 | I: 12.4 (1.7)  C:12.3 (1.7) | Diabetes duration: ≥1 year  Age: 8-16∙9 years  HbA1c: ≥47-86 mmol/mol, 6.5% & ≤10.0% | I: 4.8 (3.3)  C: 4.8 (3.2) | 6 | Family intervention (MI); counselling; face to face; family; research assistant | Care ambassador; attention control |
| Serlachius, 2016 (S47) | 147 | I: 14.36 (1.07)  C: 14.31 (1.12) | Age: 13-16 years | I: 5.63 (3.33)  C: 6.12 (3.80) | 5 | The Best of Coping; CBT; face to face; group; health psychologist | Usual care |
| Mackey, 2016 (S36) | 30 | All: 4.49 (1.71) | Diabetes duration: ≤6 months  Age: 1-6 years | All: 0.23 (0.10) | 9 | Telephone counselling; counselling; telephone; individual; telephone counsellor and parent peer | Physical activity advice; attention control |
| Wei, 2018 (S43) | 85 | I: 8.4 (1.5-14.1)  C: 8.2 (1.6-14.4)  [median, range] | Diabetes duration: ≥1 year  Age: 11-16 years | I: 4.6 (1.2-14.5)  C: 5.7 (1.6-12.9)  [median, range] | 8 | CBT; CBT; face to face; individual; therapist | Non-directive supportive counselling; attention control |
| **Studies included in systematic review only** | | | | | | | |
| Holmes, 2014 (S31) | 40 | All: 57.0 (9) | Diabetes duration: ≥1 year  Age: 11-14 years | Not reported | 4 | CST; CBT; face to face; family; family interventionist | Diabetes education; attention control |
| Wysocki, 2006 (S44) | 104 | I: 13.9 (1.9)  C1: 14.2 (1.9)  C2: 14.4 (1.9) | Diabetes duration: ≥2 years  HbA1c: ≥ 64 mmol/mol, 8% | I: 5.1 (3.0)  C1: 5.9 (4.0)  C2: 5.5 (3.2) | 12 | BFST; family systems therapy; face to face; family; psychologists | 1. Usual care 2. Educational support; attention control |
| Ellis, 2012 (S27) | 146 | All: 14.2 (2.3) | Diabetes duration: ≥1 year  Age: 10-18 years  HbA1c: ≥ 64 mmol/mol, 8% | All: 4.7 (3.0) | Variable | MST; family therapy; face to face; family; therapist | Telephone support; attention control |

MI=motivational interviewing; MST=multi-systemic therapy; CST=coping skills training; CBT=cognitive behavioural therapy; GSD= guided self-determination; BFST-D= behavioural family systems therapy for diabetes

**Table S4.** Case definition of type 1 diabetes adult studies included in meta-analysis

| **Year, Country, reference** | **Mean age (SD or range), years intervention group** | **Mean age (SD or range), years control group** | **Mean (SD or range) duration of diabetes, years intervention group** | **Mean (SD or range) duration of diabetes, years control group** | **Mean (SD) baseline HbA1c intervention group (mM, %)** | **Mean (SD or range) baseline HbA1c control group (mM, %)** | **Age inclusion criteria (years)** | **Diabetes duration inclusion criteria (months)** | **HbA1c inclusion criteria, (mM, %)** |
| --- | --- | --- | --- | --- | --- | --- | --- | --- | --- |
| Amsberg et al, 2009, Sweden, S1 | 41.1 (11.70 | 41.4 (12.9) | 19.9 (9.4) | 23.2 (11.8) | NR, 8.5% (0.9) | NR, 8.5% (0.8) | 18-65 | >2 years | 58, 7.5% or more |
| Ismail et al, 2008, UK, S2 | 36.6 (29.9) | 36.0 (25.8) | NR | NR | NR, 9.25% (8.6-10.3) | NR, 9.4 (8.8-10.2) | 18-65 | >2 years | >66, 8.2% or more |
| Snoek et al, 2008, The Netherlands, S6 | 38.1 (1.3) | 37.4 (11.1) | 17.8 (10.1) | 18.8 (10.9) | NR, 8.8% (1.3) | NR, 9.1 (1.1) | NR | >1 year | 64, >/= 8% |
| Hermanns eta l, 2015, Germany, S12 | 43.2 (14.9) | 43.4 (13.8) | 11.3 (3.0) | 14.2 (10.7) | NR, 8.9% (1.8) | NR, 8.9 (1.8) | 18-70 | NR | NR |
| Zoffmann et al, 2015, Denmark, S9 | 25.9 (5.0) | 25.3 (5.2) | 13.8 (6.9) | 13.7 (6.8) | NR, 9.5% (1.3) | NR, 9.7 (1.8) | 8-35 | >1 year | >/=64, 8% |
| Van Son et al, 2014, S20 | 56 (13) | 57 (13) | NR | NR | 59.0/7.5 (12.6/1.2) | 59.2/7.7 (13.0/1.2) | NR | NR | NR |
| Petrak et al, 2015, Germany, S16 | 49 (10.6) | 47 (12.8) | 15.7 (10.4) | 15.0 (10.6) | 78/9.3 (7.21/1.49) | 77/9.2 (1.44/7.76) | 21-69 | NR | 58, 7.5% |
| Mohn et al, 2017, Norway, S4 | 37.2 (10.9) | 36.9 (9.4) | 20.6 (11.2) | 18.5 (10.6) | 78/9.3 (12.7/1.2) | 76/9.1(10.4/1.0) | 18-55 | >1 year | >/= 64, 8% |
| Menting et al, 2017, The Netherlands, S3 | 44.4 (12.1) | 42.9 (12.5) | 24.2 (13.3) | 24.1 (13.9) | 65/8.1 (11/1.0) | 64/8.0 (11/1.0) | 18-70 | >1 year | NR |

**Table S5.** Primary outcome of type 1 diabetes adult studies included in the meta-analysis

| **Reference** | **Primary outcome category** | **Primary outcome description** |
| --- | --- | --- |
| Amsberg et al, 2009, S1 | HbA1c | HbA1c |
| Ismail et al, 2008, S2 | HbA1c | HbA1c |
| Snoek et al, 2008, S6 | HbA1c | HbA1c |
| Hermanns eta l, 2015, S12 | Psychological | Depressive symptoms |
| Zoffmann et al, 2015, S9 | HbA1c | HbA1c |
| Van Son et al, 2014, S20 | Psychological | Emotional distress |
| Petrak et al, 2015, S16 | HbA1c | HbA1c |
| Mohn et al, 2017, S4 | HbA1c | HbA1c |
| Menting et al, 2017, S3 | Biomedical | Fatigue |

**Table S6.** Case definition of type 1 diabetes children/adolescent studies included in the meta-analysis

| **Year, Country, reference** | **Mean age (SD or range), years intervention group** | **Mean age (SD or range), years control group** | **Mean (SD or range) duration of diabetes, years intervention group** | **Mean (SD or range) duration of diabetes, years control group** | **Mean (SD) baseline HbA1c intervention group (mmol/mol, %)** | **Mean (SD) baseline HbA1c control group (mmol/mol, %)** | **Age inclusion criteria (years)** | **Diabetes duration inclusion criteria (months)** | **HbA1c inclusion criteria (mmol/mol, %)** |
| --- | --- | --- | --- | --- | --- | --- | --- | --- | --- |
| Graue, 2005, Norway, S45 | 14.5 (1.6) | 14.3 (1.6) | 6.7 (3.3) | 6.9 (4.3) | NR, 9.6% (1.3) | NR, 9.4% (1.7) | 11-17 | NR | NR |
| Channon, 2007, UK, S25 | 15.3 90.97) | 15.4 (1.19) | 9.2 (1.96) | 9.1 (1.47) | NR, 9.3% (2.11) | NR, 9.0% (1.56) | 14-17 | >1 year | NR |
| Ellis, 2007, USA, S28 | 13.4 (1.9) | 13.1(2.0) | NR | NR | NR, 11.4% (2.25) | NR, 11.29% (2.34) | 10-17 | >1 year | >/= 64, 8% |
| Nansel, 2007, USA, S39 | 13.6 (1.9) | 13.9 (1.6) | 7.5 (3.4) | 7.8 (4.0) | NR | NR | 11-16 | >1 year | NR |
| Grey, 2009, USA, S29 | 9.9 (1.5) | 9.9 (1.4) | 3.7 (2.78) | 3.6 (3.0) | NR, 6.98% (1.33) | NR, 7.11% (1.21) | 8-12 | >6 months | NR |
| Wang, 2010, USA, S42 | 15.3 (1.4) | 15.6 (1.7) | 6.7 (3.4) | 7.6 (4.7) | NR, 10.9% (0.4) | NR, 11.1% (0.3) | 12-18 | >1 year | >/= 74, 9% |
| Lehmkuhl, 2010, USA, S35 | 13.72 (2.67) | 13.43 (2.17) | NR | NR | NR, 10.81% (2.05) | NR, 10.37% (1.88) | 9-17 | NR | NR |
| Robling, 2012, UK, S41 | 10.4 (2.8) | 10.7 (2.8) | 5.2 (2.8) | 5.0 (2.7) | NR, 9.4% (1.7) | NR, 9.2% (1.8) | 4-15 | >1 year | NR |
| Sassmann, 2012, Germany, S46 | 6.4 (2.3) | 5.8 (1.9) | 2.6 (1.6) | 2.6 (1.9) | NR, 7.2% (0.8) | NR, 7.1% (0.4) | 2-10 | NR | NR |
| Nansel, 2012, USA, S38 | 12.5 (1.8) | 12.4 (1.7) | 4.8 (3.3) | 4.9 (3.2) | NR, 8.4% (1.2) | NR, 8.3% (1.1) | 9-14.9 | >3 years | >42, 6%, <108, 12% |
| Najmi, 2012, Iran, S37 | 14.1 (1.8) | 15.3 (1.8) | NR | NR | NR, 8.4% (2.41) | NR, 8.49% (1.47) | 12-18 | >1 year | NR |
| Husted, 2014, Denmark, S32 | 14.9 (1.5) | 14.6 (1.3) | 6.1 (3.0) | 5.3 (3.4) | 79.9/9.5 (16.6/3.0) | 72.8/8.8 (9.4/3.0) | 13-18 | >1 year | >/= 64, 8% |
| Jaser, 2014, USA, S33 | 15.3 (1.4) | 15.0 (1.6) | 7.3 (4.3) | 6.5 (3.5) | NR, 8.5% (1.1) | NR, 8.2% (1.0) | 13-17 | >6 months | NR |
| Katz, 2014, USA, S34 | 12.7 (2.2) | 12.5 (2.30 | 6.5 (3.8) | 5.7 (3.5) | NR, 8.4% (1.4) | NR, 8.4% (1.3) | 8-16 | >/=6 months | NR |
| Christie, 2014, UK, S26 | 13.1 (2.1) | 13.2 (2.1) | 5.7 (3.2) | 6.1 (3.3) | NR, 9.9% (1.5) | NR, 10.0% (1.5) | 8-16 | >1 year | >/= 68, 8.5% |
| Harris, 2015, S30 | 15.04 (1.79) | 14.94 (1.77) | 6.51 (3.24) | 6.56 (3.77) | 97.8 /11.10 (18.56/2.14) | 98.4/11.15 (15.27/1.73) | 12-18 | >1 year | >/=74.9. 9% |
| Nansel, 2015, S40 | 12.6 (2.7) | 13.0 (2.5) | 5.6 (2.5) | 6.3 (3.6) | NR, 8.1% (1.1) | NR, 8.1% (1.0) | 8.0-16.9 | >1 year | >/=48, 6.5%, </= 86, 10.0% |
| Serlachius, 2016, S47 | 14.36 (1.07) | 14.31 (1.12) | 5.63 (3.33) | 6.12 (3.80) | NR, 8.49% (1.48) | NR, 8.60% (1.38) | 13-16 | NR | NR |
| Mackey, 2016, S35 | All: 4.49 (1.71) |  | All: 0.23 (0.10) |  | All: NR, 8.28% (0.96) |  | 1-6 | <6 months | NR |
| Wei, 2018, S43 | Median: 8.4 (1.4-4.1) | Median: 14.1 (11.7-16.6) | Median: 4.6 (1.2-14.5) | Median: 5.7 (1.6-12.9) | 77/9.2 (55-134/ 7.2-14.4) | 73/8.8 (44-132/6.2-14.2) | 11-16 | >1 year | NR |

**Table S7.** Primary outcome of type 1 diabetes child and adolescent studies included in meta-analysis

| **Reference** | **Primary outcome category** | **Primary outcome description** |
| --- | --- | --- |
| Graue, 2005, Norway, S45 | HbA1c | HbA1c |
| Channon, 2007, UK, S25 | HbA1c | HbA1c |
| Ellis, 2007, USA, S28 | HbA1c | HbA1c |
| Nansel, 2007, USA, S39 | HbA1c | HbA1c |
| Grey, 2009, USA, S29 | HbA1c | HbA1c |
| Wang, 2010, USA, S42 | HbA1c | HbA1c |
| Lehmkuhl, 2010, USA, S35 | HbA1c | HbA1c |
| Robling, 2012, UK, S41 | HbA1c | HbA1c |
| Sassmann, 2012, Germany, S46 | Parenting skills | Parenting behaviour |
| Nansel, 2012, USA, S38 | HbA1c | HbA1c |
| Najmi, 2012, Iran, S37 | HbA1c | HbA1c |
| Husted, 2014, Denmark, S32 | HbA1c | HbA1c |
| Jaser, 2014, USA, S33 | Self-management | Self-efficacy |
| Katz, 2014, USA, S34 | HbA1c | HbA1c |
| Christie, 2014, UK, S26 | HbA1c | HbA1c |
| Harris, 2015, S30 | HbA1c | HbA1c |
| Nansel, 2015, S40 | Self-management | Dietary behaviour |
| Serlachius, 2016, S47 | HbA1c | HbA1c |
| Mackey, 2016, S46 | HbA1c | HbA1c |
| Wei, 2018, S43 | HbA1c | HbA1c |

**Table** **S8.** Direct and indirect treatment effects (where indirect treatment effects were available) and the difference between them, including significance test for difference in studies for adults with type 1 diabetes.

| Treatment comparison | Side | Direct |  | Indirect |  | Difference | |  |
| --- | --- | --- | --- | --- | --- | --- | --- | --- |
|  |  | b | SE | b | Se | b | SE | p |
| CBT | Usual care | 0.25 | 0.108 | 0.268 | 0.275 | -0.014 | 0.295 | 0.96 |
| CBT | Counselling | 0.19 | 0.131 | 0.047 | 0.164 | 0.143 | 0.210 | 0.50 |
| *CBT | Attention control | -0.20 | 0.142 | 0.516 | 44.733 | -0.716 | 44.733 | 0.99 |
| CBT | Waiting list | 0.21 | 0.145 | 0.286 | 0.191 | -0.074 | 0.240 | 0.76 |
| Counselling | Usual care | 0.13 | 0.106 | 0.083 | 0.283 | 0.045 | 0.302 | 0.88 |
| Counselling | Waiting list | 0.13 | 0.151 | 0.060 | 0.186 | 0.074 | 0.240 | 0.76 |

**The large SE of 44.73 for the comparison between CBT and attention control suggests an unidentified model and conclusion about homogeneity needs to be treated with care.*

**Table S9. Summary of pairwise comparisons of all treatment assuming common heterogeneity estimate for all treatment design comparisons for adults with type 1 diabetes. SMD=Standardised mean difference**

| Treatment comparison | | SMD | (95% C.I>) | SE | z | p |
| --- | --- | --- | --- | --- | --- | --- |
| CBT | Counselling | 0·134 | (-0.067 to 0.334) | 0·102 | 1·31 | 0·19 |
| CBT | Usual care | 0·256 | (0.059 to 0.452) | 0·1 | 2·55 | 0·011 |
| CBT | Attention control | -0·200 | (-0.479 to 0.078) | 0·142 | -1·41 | 0·16 |
| CBT | Waiting list | 0·239 | (0.013 to 0.465) | 0·115 | 2·07 | 0·038 |
| Counselling | Usual care | 0·122 | (-0.071 to 0.316) | 0·099 | 1·24 | 0·22 |
| Counselling | Attention control | -0·334 | (-0.677 to 0.01) | 0·175 | -1·91 | 0·057 |
| Counselling | Waiting list | 0·105 | (-0.124 to 0.335) | 0·117 | 0·9 | 0·37 |
| Usual care | Attention control | -0·456 | (-0.797 to -0.115) | 0·174 | -2·62 | 0·009 |
| Usual care | Waiting list | -0·017 | (-0.281 to 0.247) | 0·135 | -0·13 | 0·9 |
| Attention control | Attention control | -0·440 | (-0.798 to -0.08) | 0·183 | -24 | 0·016 |

**Table S10.** Direct and indirect treatment effects (where indirect treatment effects were available) and the difference between them, including standard errors (SE) and significance test for difference in studies for children and adolescents with type 1 diabetes.

| Treatment comparison | | Direct |  | Indirect |  | Difference | |  |
| --- | --- | --- | --- | --- | --- | --- | --- | --- |
|  |  | b | SE | b | SE | b | SE | p |
| CBT | Family therapy | -0.091 | 0.705 | -0.189 | 1.562 | 0.098 | 1.714 | 0.95 |
| CBT | Usual care | 0.041 | 0.565 | 0.877 | 0.771 | -0.836 | 0.956 | 0.38 |
| CBT | Attention control | 0.381 | 0.573 | -0.624 | 0.848 | 1.004 | 1.023 | 0.33 |
| CBT | Waiting list | 0.476 | 1.049 | 0.574 | 1.356 | -0.098 | 1.714 | 0.95 |
| Counselling | Usual care | 0.059 | 0.482 | -0.944 | 0.901 | 1.003 | 1.022 | 0.33 |
| Counselling | Attention control | -0.629 | 0.454 | 0.376 | 0.917 | -1.005 | 1.023 | 0.33 |
| Family therapy | Waiting list | 0.32 | 1.05 | 0.222 | 1.355 | 0.098 | 1.715 | 0.95 |

**Table S11.** Summary of all pairwise comparisons of treatment effects assuming common heterogeneity estimate for all treatment design comparisons for children with type 1 diabetes. SMD=standardised mean difference.

| Treatment comparison | | SMD | (95% C.I>) | SE | z | p |
| --- | --- | --- | --- | --- | --- | --- |
| CBT | Counselling | 0.496 | (-0.49 to 1.481) | 0.503 | 0.99 | 0.32 |
| CBT | Usual care | 0.332 | (-0.54 to 1.204) | 0.445 | 0.75 | 0.46 |
| CBT | Attention control | 0.065 | (-0.849 to 0.979) | 0.466 | 0.14 | 0.89 |
| CBT | Family therapy | 0.225 | (-1.153 to 1.604) | 0.703 | 0.32 | 0.75 |
| CBT | Waiting list | 0.511 | (-1.033 to 2.055) | 0.788 | 0.65 | 0.52 |
| Counselling | Usual care | -0.165 | (-0.989 to 0.659) | 0.421 | -0.39 | 0.70 |
| Counselling | Attention control | -0.435 | (-1.224 to 0.354) | 0.403 | -1.08 | 0.28 |
| Counselling | Family therapy | -0.271 | (-1.697 to 1.154) | 0.727 | -0.37 | 0.71 |
| Counselling | Waiting list | 0.013 | (-1.707 to 1.733) | 0.877 | 0.01 | 0.99 |
| Usual care | Attention control | -0.267 | (-1.239 to 0.706) | 0.496 | -0.54 | 0.59 |
| Usual care | Family therapy | -0.106 | (-1.298 to 1.085) | 0.608 | -0.17 | 0.86 |
| Usual care | Waiting list | 0.179 | (-1.41 to 1.767) | 0.81 | 0.22 | 0.83 |
| Attention control | Family therapy | 0.163 | (-1.331 to 1.657) | 0.762 | 0.21 | 0.83 |
| Attention control | Waiting list | 0.447 | (-1.282 to 2.176) | 0.882 | 0.51 | 0.61 |
| Family therapy | Waiting list | 0.285 | (-1.258 to 1.828) | 0.787 | 0.36 | 0.72 |


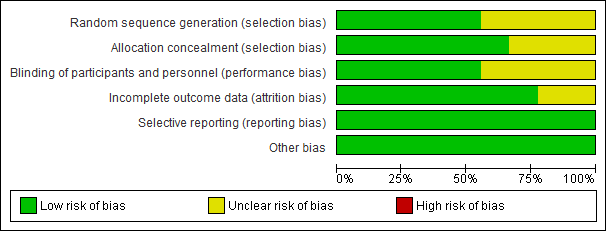

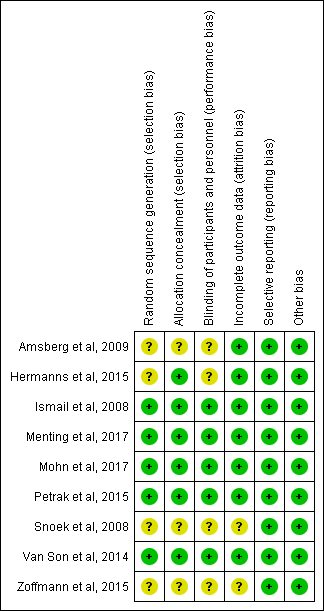


**Figure S1- Risk of bias domain assessment across studies for adults with type 1 diabetes.**

**Figure S2- Risk of bias within studies for adults with type 1 diabetes**


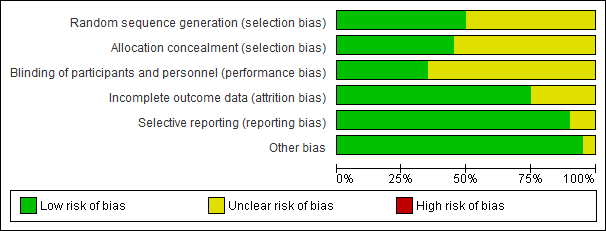


**Figure S3- Risk of bias domain assessment across studies for children/adolescents with type 1 diabetes.**


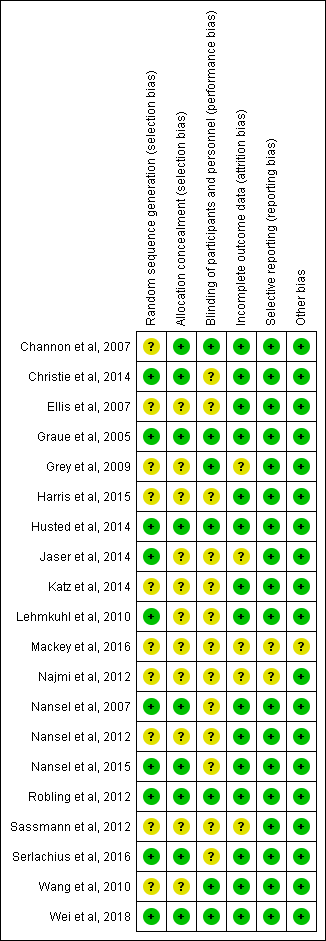


**Figure S4- Risk of bias within studies for children/adolescents with type 1 diabetes.**


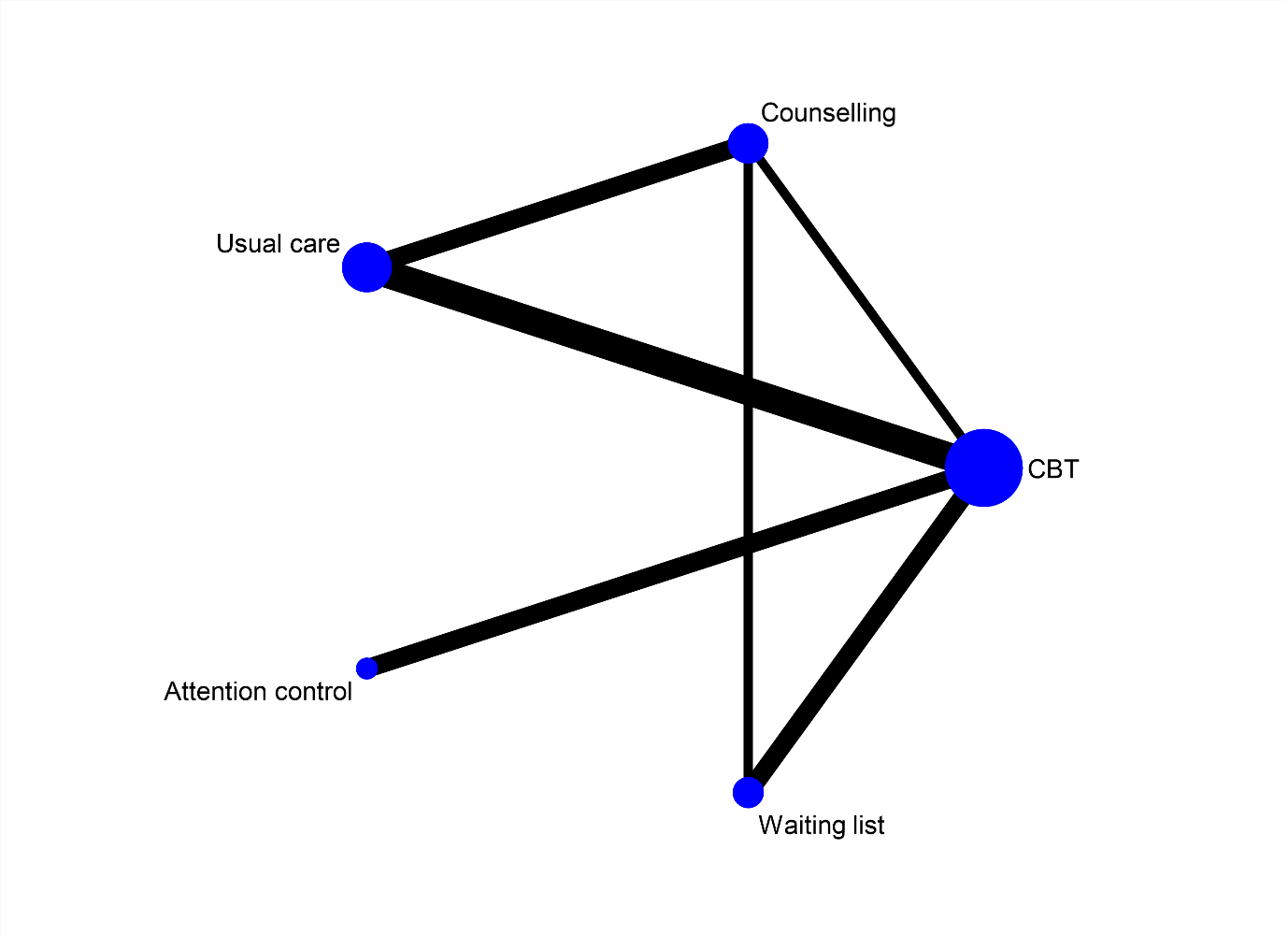


**Figure S5*.* Network plots of direct comparisons for the network meta-analysis for adults with type 1 diabetes.**

The width of the lines is proportional to the number of trials comparing each pair of treatments and the size of each node is proportional to the number of studies testing the specific treatment. It shows roughly how much information is available for each treatment and for each treatment comparison.


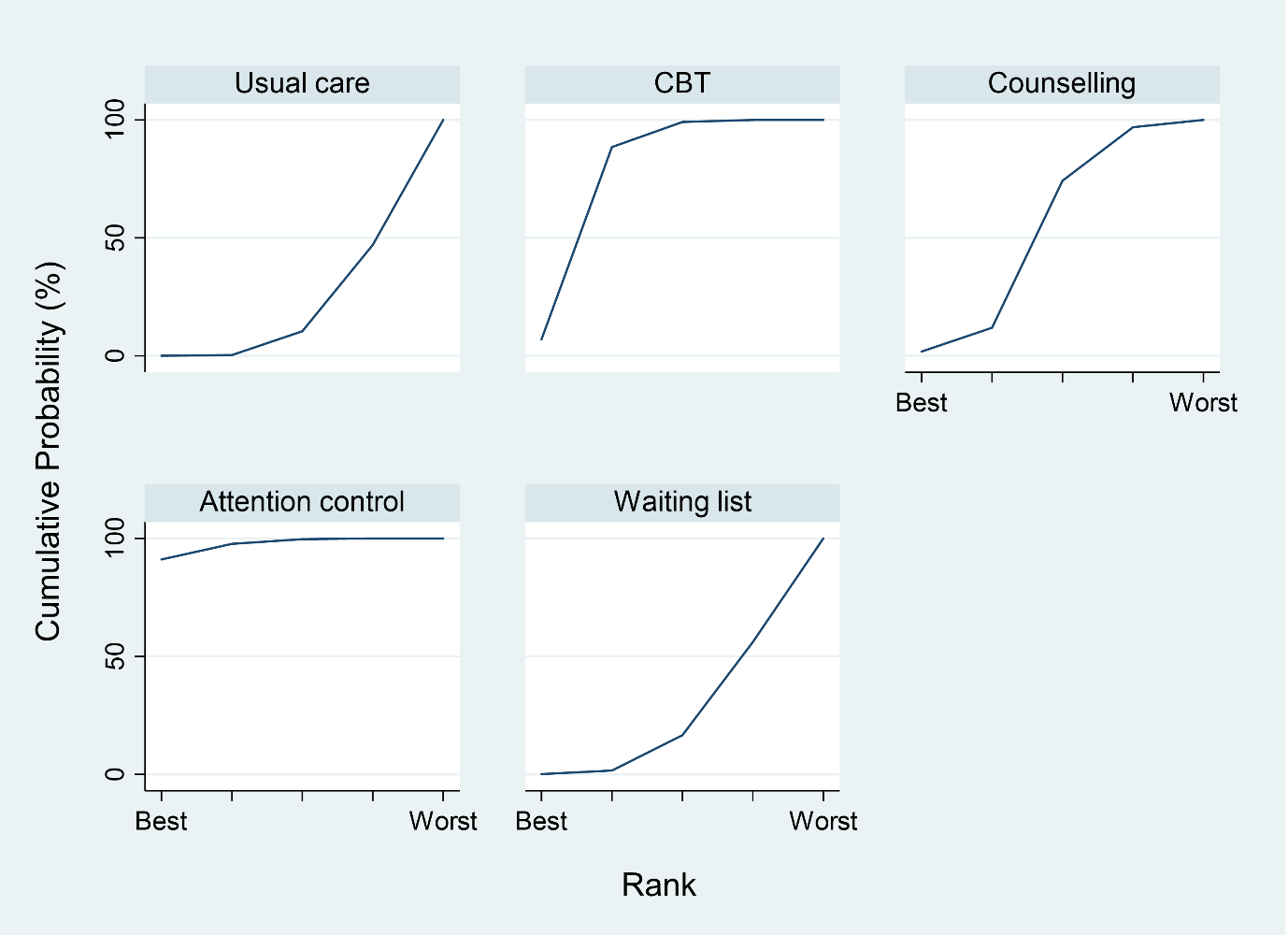


**Figure 6. Rankogram for all treatments for adults with type 1 diabetes.**

The plot shows the surface under the cumulative ranking curves for all treatments. For example, usual care has a very low probability to be among the best treatments but a very high to be one of the worst.


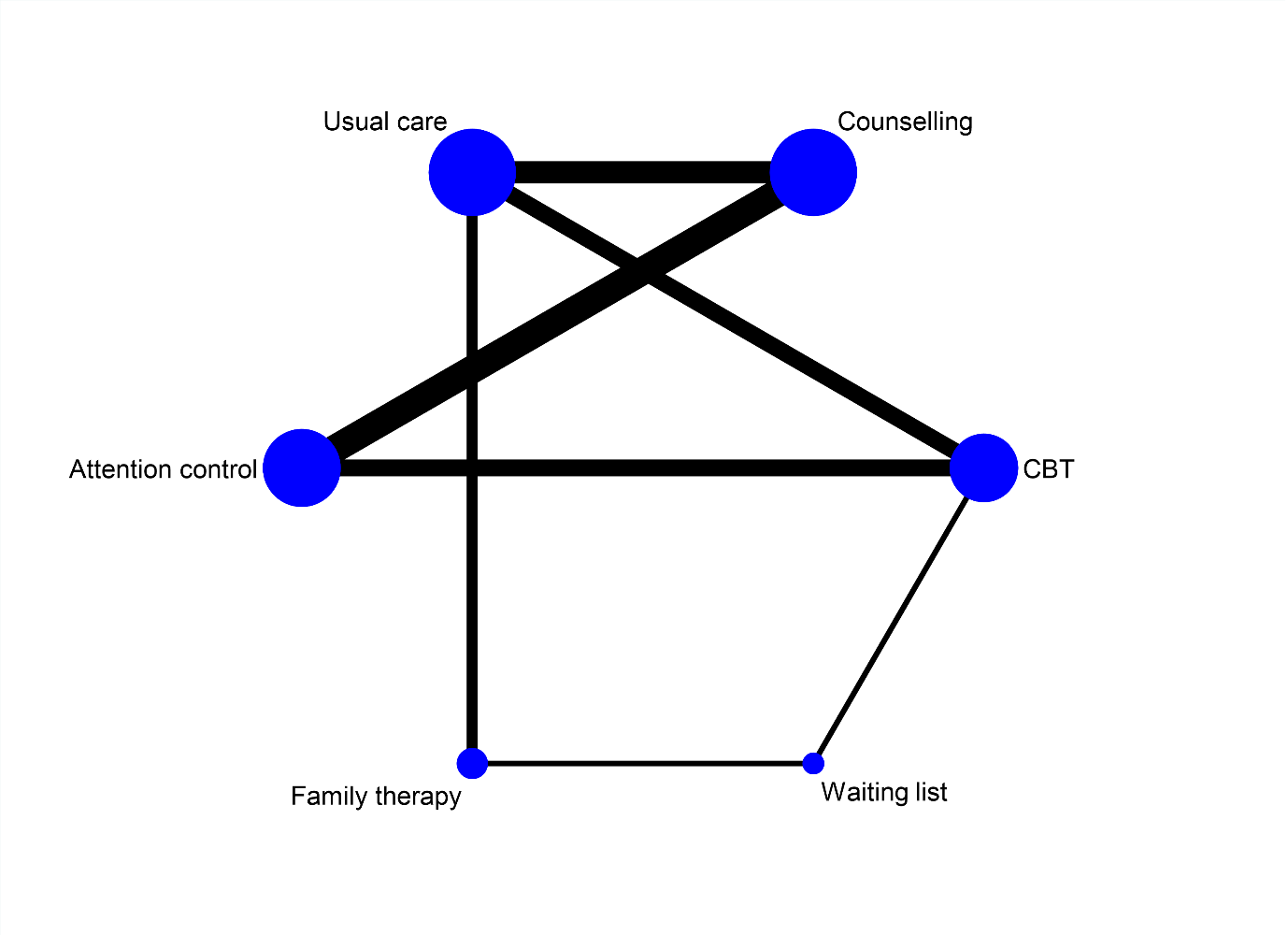


**Figure S7. Network plots of direct comparisons for the network meta-analysis for children and adolescents with type 1 diabetes.**

The width of the lines is proportional to the number of trials comparing each pair of treatments and the size of each node is proportional to the number of studies testing the specific treatment. It shows roughly how much information is available for each treatment and for each treatment comparison.


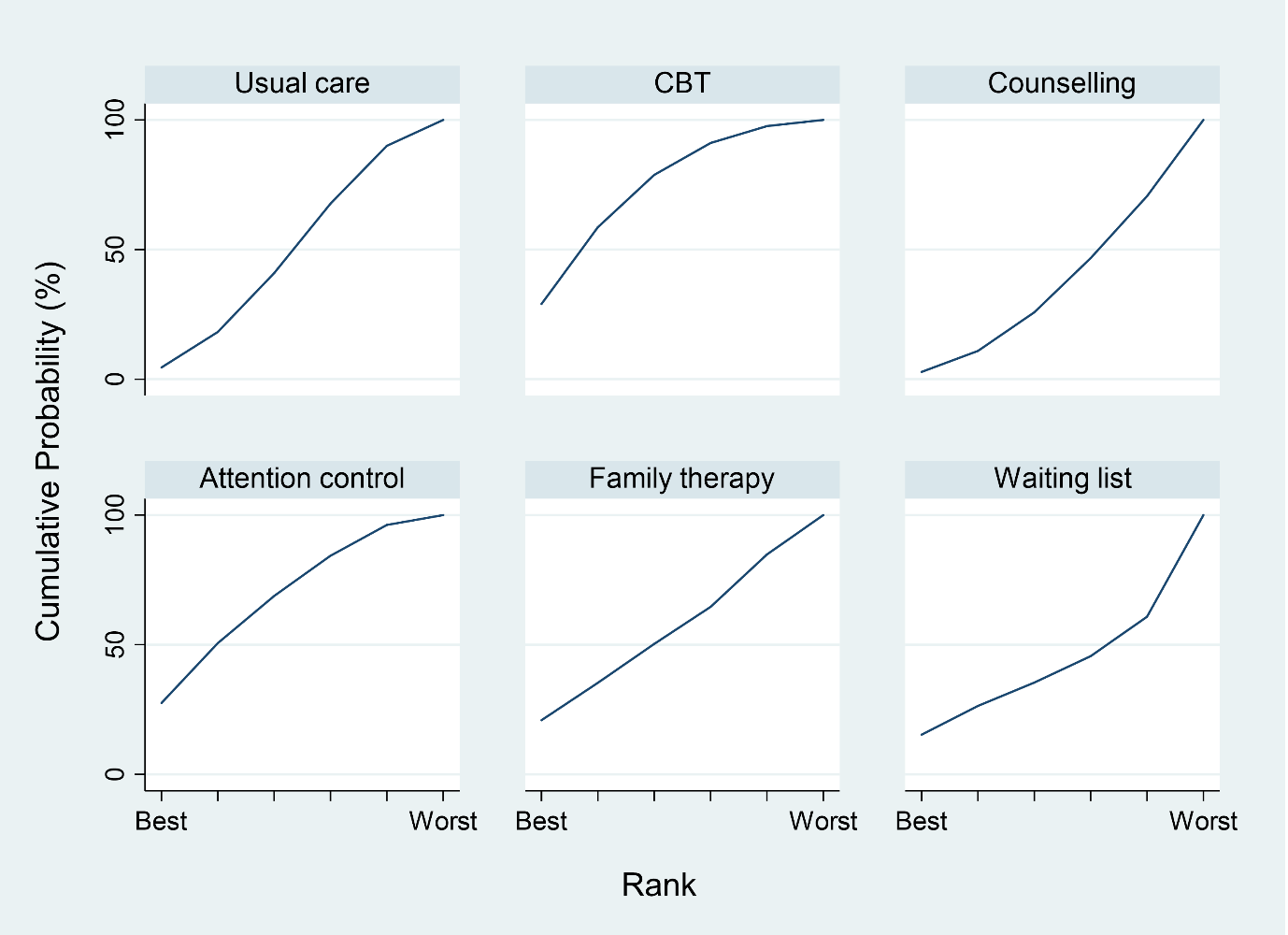


**Figure S8. Rankogram for all treatments for children and adolescents with type 1 diabetes.**

The plot shows the surface under the cumulative ranking curves for all treatments. For example, usual care has a very low probability to be among the best treatments but a very high to be one of the worst.
